# Pharmacological Effects and Mechanisms of Tanshinone IIA in Bone Injury Repair

**DOI:** 10.3390/ph18091338

**Published:** 2025-09-05

**Authors:** Weijian Hu, Yameng Si, Xinru Wen, Duan Lin, Zihao Yu, Xin Xie, Jiabin Xu

**Affiliations:** 1 Medical College, Northwest University, Xi’an 710127, China; 202324229@stumail.nwu.edu.cn; 2 School of Stomatology, Xuzhou Medical University, Xuzhou 221004, China; 18905200972@189.cn; 3 College of Life Sciences, Northwest University, Xi’an 710127, China; 18082202290@163.com (X.W.); 202332731@stumail.nwu.edu.cn (D.L.);

**Keywords:** Tanshinone IIA, bone injury, signaling pathways, anti-inflammatory, angiogenesis, multiorgan-protection, pharmacokinetics, multi-target effects

## Abstract

Tanshinone IIA (T-IIA), a fat-soluble diterpene quinone extracted from *Salvia miltiorrhiza*, is widely recognized for its multiple pharmacological properties, including anti-inflammatory, antioxidant, anti-fibrotic, and anti-tumor effects. Recent studies have highlighted its great potential in treating bone metabolic disorders, especially osteoporosis and bone damage repair. Bone health depends on the dynamic balance between osteoblast-mediated bone formation and osteoclast-mediated bone resorption. Disruption of this balance can lead to diseases such as osteoporosis, which is often diagnosed after a fracture, seriously affecting the quality of life and increasing the medical burden. Early identification of high-risk groups and appropriate treatment are essential for preventing fracture recurrence. Studies have shown that T-IIA can promote osteoblast differentiation and inhibit osteoclast activity, targeting key signaling pathways such as NF-κB, PI3K/Akt, and Wnt/β-catenin, all of which are closely related to bone metabolism. T-IIA has a dual role in regulating bone formation and bone resorption, making it a potential drug for the treatment of osteoporosis. In addition, T-IIA has neuroprotective, hepatic, renal, cardiac, and cerebral effects, which enhance its therapeutic effect. Despite the remarkable efficacy of T-IIA, its clinical application is limited due to poor solubility and low bioavailability. Recent advances in drug delivery systems, such as liposome formulations and nanocarriers, have improved their pharmacokinetics, increased absorption rate, and bioavailability. Combination therapy with growth factors or stem cells can further enhance its efficacy. Future studies should focus on optimizing the delivery system of T-IIA and exploring its combined application with other therapeutic strategies to expand its clinical application range.

## 1. Introduction

### 1.1. Overview of Bone Injury

Bone is a dynamic tissue that undergoes continuous remodeling and its structural integrity relies on a balance between bone formation and bone resorption. Osteoblasts mediate bone formation by synthesizing and depositing new bone matrix, whereas osteoclasts are responsible for degrading and resorbing mineralized bone. In healthy individuals, bone remodeling remains in dynamic equilibrium. When this balance is disrupted—especially by impaired osteogenesis or excessive resorption—it leads to reduced bone mass and microarchitectural damage, eventually causing osteoporosis [1]. Osteoporosis is among the most prevalent metabolic bone diseases globally. Its early clinical presentation is often subtle and insidious, and diagnosis frequently occurs only after a low-energy fracture has occurred. Fractures not only severely diminish a patient’s quality of life but also impose a heavy economic burden on healthcare systems. In individuals over 50, an initial fracture is a strong predictor of subsequent fractures, with the risk increasing sharply in the first year post-fracture. Although many patients regard fractures as isolated accidents, clinicians should treat them as warning signs of underlying osteoporosis, even if a fracture results from high-energy trauma. Among osteoporosis-related fractures, vertebral fractures are especially common, including asymptomatic subclinical vertebral fractures detected only by imaging. Such fractures can increase the risk of hip fracture by nearly five-fold and other non-spinal fractures by approximately 2–3-fold [2]. Without timely intervention, patients may enter a vicious cycle of repeated fractures and progressive mobility limitation, potentially leading to severe disability or even premature death.

Given the profound impact of fractures on patient independence, quality of life, and longevity, accurately identifying and proactively managing high-risk individuals has become a core priority in osteoporosis prevention strategies. However, despite comprehensive diagnostic and treatment guidelines, real-world practice still shows a marked deficit in primary prevention. Studies indicate that even after a fracture (in the secondary prevention phase), about 80–90% of high-risk patients fail to receive appropriate therapeutic interventions [3]. First-line treatments currently consist mainly of anti-resorptive agents such as alendronate, denosumab, risedronate, and zoledronic acid, which have proven efficacy in reducing vertebral, hip, and other non-spinal fracture incidence. However, these drugs share pharmacokinetic limitations (e.g., prolonged retention in the body, slow clearance, and low ~1–3% oral bioavailability), which constrain their use in some patient populations [4]. Abundant evidence shows that implementing proper anti-fracture therapy in eligible patients can significantly reduce refracture risk and improve outcomes. In this context, developing novel therapeutic strategies with high target specificity, favorable safety profiles, and broad regulatory effects on bone metabolism via multiple pathways has become a research focus. In recent years, active compounds derived from natural products—owing to their complex chemical structures, diverse biological functions, and the ability to modulate multiple pathways synergistically—have attracted considerable attention in the field of osteoporosis prevention. Numerous studies demonstrate that natural medicines have notable advantages in promoting osteogenic differentiation, inhibiting bone resorption, and modulating bone metabolic pathways. It is anticipated that such bioactive natural substances could serve as alternative or adjunct therapies within the current osteoporosis treatment paradigm, thereby expanding personalized therapeutic strategies for bone metabolic diseases.

Bone injuries such as fractures, delayed unions, and non-unions remain common clinical challenges with significant treatment difficulty. Particularly in conditions like delayed fracture healing, non-union, osteoporotic fractures, and impaired bone regeneration, there is a pressing need for improved strategies to repair bone tissue. In recent years, increasing research has focused on natural products for bone repair and regeneration. Among these, Tanshinone IIA, a bioactive diterpene quinone constituent of *Salvia miltiorrhiza* (Danshen), has emerged as a promising candidate due to its wide availability, well-defined structure, and potent biological activities. As a traditional Chinese medicinal herb, *Salvia miltiorrhiza* (Danshen) has long been used clinically to promote blood circulation, remove blood stasis, nourish blood, and calm the mind. Modern pharmacological and clinical studies have confirmed that Danshen exerts significant therapeutic effects against various systemic diseases, including cardiovascular and cerebrovascular diseases, neurodegenerative disorders, hepatic and renal dysfunction, gynecological diseases, and bone metabolic disorders [5]. Tanshinone IIA, one of the most representative lipophilic diterpene quinones in Danshen, is characterized by a stable molecular structure and high lipophilicity. With its broad spectrum of pharmacological actions and multiple molecular targets, Tanshinone IIA has become an important candidate for drug development in natural product research.

### 1.2. Pharmacological Potential and Research Value of Tanshinone IIA

Studies have shown that Tanshinone IIA modulates numerous physiological processes, with reported activities including anti-inflammatory, antioxidant, metabolic regulatory (glucose and lipid metabolism), anti-fibrotic, antithrombotic, and cardioprotective effects. It has been widely studied in basic and translational research models of atherosclerosis, metabolic syndrome, and other chronic inflammation-related diseases [6]. In cardiovascular disease models, Tanshinone IIA promotes angiogenesis and improves endothelial function, effectively slowing the progression of conditions such as atherosclerosis and hypertension. Mechanistically, these effects mainly involve modulation of inflammatory and oxidative stress signaling pathways. Notably, Tanshinone IIA exerts anti-inflammatory, anticoagulant, and cardioprotective effects by inhibiting NF-κB activation through the TLR and MAPK signaling pathways. Furthermore, Tanshinone IIA suppresses excessive immune cell activation and the release of inflammatory mediators, helping maintain immune homeostasis. In nervous system disorders, Tanshinone IIA has been shown to attenuate neuroinflammatory responses and neuronal apoptosis. Specifically, it reduces neuroinflammation and protects neurons by modulating the MAPK pathway (downregulating COX-2 and prostaglandin levels) and by suppressing TLR2/NF-κB signaling, thereby exerting significant neuroprotective effects in central inflammatory conditions. In addition, Tanshinone IIA’s anti-tumor activity is closely tied to its regulation of key signaling pathways such as p53 and AKT/mTOR, and it has demonstrated the potential to inhibit tumor growth in various solid tumor models [7]. In metabolic disease contexts, Tanshinone IIA’s effects on diabetes and its complications have also garnered attention. In streptozotocin (STZ)-induced diabetic rats, Tanshinone IIA significantly reduced proteinuria and kidney hypertrophy, and attenuated pathological damage in renal tissue. Its mechanisms included inhibiting malondialdehyde (MDA) production, increasing superoxide dismutase (SOD) activity, and downregulating inflammatory and fibrotic factors like MCP-1, TGF-β1, P-selectin, and C-reactive protein (CRP), collectively producing a synergistic renoprotective effect [8]. In studies of antioxidant mechanisms, a high-throughput screen targeting estrogen-related receptor γ (*ESRRG*) found that Tanshinone IIA can reduce reactive oxygen species (ROS) levels by modulating the *ESRRG/CYP2E1* signaling axis, thereby slowing age-related liver functional decline and highlighting its potential value in anti-aging interventions [9].

Importantly, research on Tanshinone IIA in the prevention and treatment of bone metabolic diseases has been especially promising. A large body of in vivo and in vitro evidence shows that Tanshinone IIA can effectively promote osteoblast differentiation and bone matrix formation while inhibiting osteoclast generation and activity, demonstrating strong anti-osteoporotic potential. This bone-protective effect is primarily attributed to Tanshinone IIA’s inhibition of NF-κB activation and the downregulation of pro-inflammatory factors that drive bone resorption. Moreover, in disease models characterized by bone destruction, such as rheumatoid arthritis, Tanshinone IIA has exhibited a bidirectional regulatory effect on bone loss [10]. Taken together, Tanshinone IIA targets multiple pathways and processes—including anti-oxidative, anti-apoptotic, metabolic regulatory, anti-angiogenic, and anti-senescent mechanisms—to exert broad pharmacological activities across various organ systems, making it a compelling natural product for further research in bone injury repair.

### 1.3. Active Constituents of Salvia miltiorrhiza: Tanshinone IIA, Related Diterpenoids, and Salvianolic Acids

**Figure 1:**
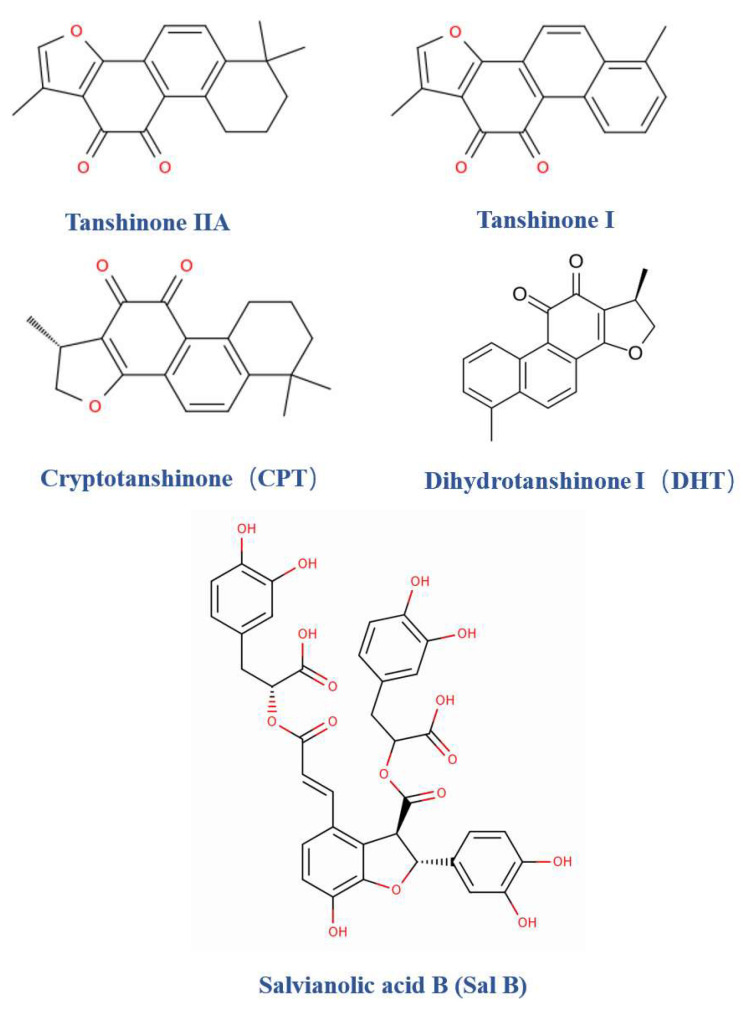
Chemical structures of the main active ingredients in Danshen.

The principal bioactive constituents of *Salvia miltiorrhiza* (Danshen) fall into two major classes. The first comprises lipophilic diterpene quinones (tanshinones)—predominantly abietane-type diterpenoids bearing o-quinone and benzofuran-like ring systems. As shown in Figure 1, representative molecules include Tanshinone IIA (Tan IIA), Tanshinone I (Tan I), cryptotanshinone (CPT), and dihydrotanshinone I (DHT). The second class consists of hydrophilic polyphenolic acids (salvianolic acids), typified by salvianolic acid B (SalB), an oligomer of caffeic acid–derived units. Multi-omics and quantitative phytochemical analyses indicate that tanshinones together with salvianolic acids constitute the core chemical families of Danshen: the former are lipophilic and readily interact with membrane and mitochondrial microenvironments, whereas the latter are hydrophilic, excel at reactive oxygen species (ROS) scavenging and endothelial protection, and display complementary tissue/polarity distributions—together underpinning a lipophilic–hydrophilic dual-channel pharmacology for Danshen [11].

Tanshinone IIA and comparison with congeners. Tanshinone IIA is the most extensively studied tanshinone and exhibits multi-pathway, multi-organ protective and disease-modifying actions. In the cardiovascular system, Tanshinone IIA and its water-soluble derivative sodium tanshinone IIA sulfonate (STS) have shown, in preclinical and clinical studies, anti-ischemia–reperfusion, anti-inflammatory, and anti-fibrotic activities, together with anti-platelet effects and improvement of microcirculation. In renal, neurological, and oncologic models, Tanshinone IIA likewise demonstrates clear mechanistic and functional efficacy. Canonical signaling features include NF-κB inhibition, Nrf2/HO-1 activation, and remodeling of the TGF-β/Smad and PI3K/Akt/MAPK axes, alongside modulation of mitochondrial homeostasis and redox balance. By comparison, CPT is particularly prominent in cancer and fibrosis research, where it produces sustained STAT3 suppression with downstream network inhibition, interferes with metabolic–inflammatory coupling and cell-cycle progression, and is widely regarded as a STAT3-oriented lead. Tan I and DHT, which share the abietane skeleton but differ in saturation and substituents, display distinct strengths in regulating mitochondrial function, ROS homeostasis, and MAPK/NF-κB signaling; in selected models, they show greater potency in anti-inflammatory, antimicrobial, or anti-angiogenic outcomes. Overall, Tanshinone IIA emphasizes cardiovascular and multi-organ protection, CPT favors transcriptional-signal suppression and tumor microenvironment modulation, and Tan I/DHT provides complementary control of inflammation and stress responses [12,13,14,15,16].

Positioning and appraisal of salvianolic acids. Owing to their high polarity and polyphenolic nature, salvianolic acids—especially SalB—exhibit potent antioxidant/ROS-scavenging, anti-inflammatory, anti-apoptotic, anti-fibrotic, and endothelium-protective (anti-platelet) properties. A coherent evidence base spans cardiovascular and cerebrovascular disease, hepatic/renal injury, skeletal and cartilaginous degeneration, and regulation of stem-cell differentiation. Mechanistically, SalB activates PI3K/Akt and JAK2/STAT3 cytoprotective pathways while attenuating mitochondrial dysfunction and inflammatory cascades. From a pharmaceutical perspective, its aqueous solubility and chemical modifiability make salvianolic acids suitable for nanoparticle formulations and local depot/controlled-release strategies. Compared with the more membrane/mitochondria-targeting tanshinones, salvianolic acids function predominantly as systemic and matrix-level homeostasis modulators; in combination regimens, the two classes frequently display marked pharmacokinetic and pharmacodynamic complementarity with synergistic effects [17,18].

Synthesis and therapeutic strategy. Current evidence supports a complementary distribution–function paradigm for Danshen constituents: lipophilic abietane diterpene quinones (e.g., Tanshinone IIA and congeners) provide signaling-level and organ-protective benefits, whereas hydrophilic salvianolic acids confer system-level antioxidant and homeostatic maintenance. This duality rationalizes the observed therapeutic breadth of Danshen and informs future strategies that combine structure refinement and delivery optimization for tanshinones with multi-component synergy leveraging salvianolic acids to achieve balanced efficacy and safety across complex indications.

## 2. Chemical Properties and Pharmacokinetics of Tanshinone IIA

### 2.1. Plasma Half-Life and Bioavailability

Extensive pharmacokinetic studies indicate that the systemic oral bioavailability of Tanshinone IIA is extremely low. Most reports show oral absorption in rats of only ~2.1–6.17%. The poor oral bioavailability is mainly due to Tanshinone IIA’s high lipophilicity, poor water solubility, and pronounced first-pass hepatic metabolism, which collectively limit its absorption [19]. To overcome these barriers, recent efforts have focused on constructing novel drug delivery systems (e.g., solid dispersions, liposomes, nanocarriers) aimed at improving Tanshinone IIA’s in vivo exposure and therapeutic efficacy [20].

The plasma half-life (t_1/2) of Tanshinone IIA is highly dependent on the route of administration. Studies have found that in rats, the plasma t_1/2 is about 21.17 ± 3.98 min after intravenous injection, whereas oral administration—due to slow absorption and broad tissue distribution—results in a significantly prolonged apparent t_1/2. Tanshinone IIA follows a multi-compartment model and distributes widely to tissues such as the liver, lungs, and kidneys, which complicates its clearance kinetics [21]. Some studies note that the t_1/2 of Tanshinone IIA in rodents is typically around 50–57 min, classifying it as a moderately short-acting compound. Its extremely low oral bioavailability (<2%) and significant first-pass effect indicate that Tanshinone IIA is not suitable as a conventional oral formulation. It likely requires injectable formulations or advanced delivery technologies to improve its pharmacokinetic behavior and clinical prospects [22].

Many studies confirm the pivotal role of formulation technology in improving Tanshinone IIA’s pharmacokinetic performance. For example, encapsulating Tanshinone IIA in lipid nanocapsules (Tanshinone IIA-LNCs) via a phase inversion method significantly increased its oral absorption and prolonged its retention time in vivo. The LNCs had a particle size of ~70 nm, PDI < 0.2, zeta potential ~−13.5 mV, extremely high encapsulation efficiency (~98%), and good drug loading (2.6 mg/g). In rats, the LNC formulation achieved an AUC_0–∞ about 3.6-fold higher than a Tanshinone IIA suspension (*p* ≤ 0.01), along with a significantly prolonged t_1/2 and mean residence time, reflecting a long-circulation profile [23]. Another study prepared a Tanshinone IIA solid dispersion using a porous silica carrier (SYLOID), greatly improving the compound’s solubility and oral bioavailability. In rats, the solid dispersion achieved a C_max of 375.24 ± 79.93 ng/mL and an AUC_0–24 of 1019.87 ± 161.82 ng·h/mL, compared to 109.2 ± 59.58 ng/mL and 343.70 ± 75.63 ng·h/mL for the free drug. Consequently, the solid dispersion increased the relative bioavailability of Tanshinone IIA to 165.36% ± 28.50%, and extended its t_1/2 from 4.54 ± 1.07 h (raw drug) to 5.50 ± 1.39 h. This improvement was attributed to Tanshinone IIA adopting an amorphous state in the dispersion and forming hydrogen bonds with the carrier, which together improved its dissolution rate and wettability, thereby enhancing in vivo performance [24]. Additionally, researchers developed a delivery system based on mesoporous silica nanoparticles coated with a biotinylated lipid bilayer (Bio-LB-MSNs) to further improve Tanshinone IIA’s transmembrane absorption and cellular uptake. In a Caco-2 cell model, this system showed superior in vitro release behavior, cellular uptake, and apparent permeability (P_app) compared to unmodified Tanshinone IIA. Consistently, in vivo pharmacokinetics demonstrated that TanIIA@Bio-LB-MSNs achieved an AUC ~3.4-fold higher than raw Tanshinone IIA, with significantly improved bioavailability. Moreover, this system lowered the IC_50 against NB4 leukemia cells to 6.5 μM, indicating excellent anti-tumor activity and good prospects for clinical development [25].

### 2.2. Drug Delivery and Formulation Advances

As the most representative active constituent of Danshen, Tanshinone IIA has been widely confirmed to possess diverse pharmacological activities such as antioxidant, anti-inflammatory, anti-fibrotic, and anti-tumor effects. However, its clinical use is still limited by multiple pharmacokinetic obstacles, including poor water solubility, low oral absorption, limited bioavailability, and rapid in vivo clearance. To overcome these challenges, extensive research in recent years has been devoted to redesigning Tanshinone IIA delivery systems and optimizing formulation techniques to improve its in vivo pharmacokinetic profile and efficacy. Various nanotechnology-based delivery platforms have been developed to enhance Tanshinone IIA’s pharmacokinetic behavior. Among them, liposomes, solid lipid nanoparticles (SLNs), polymeric nanoparticles, and nanocapsules have been shown not only to significantly improve Tanshinone IIA’s stability and encapsulation efficiency but also to effectively slow its metabolic clearance, thereby increasing systemic exposure and prolonging the duration of its pharmacological effects. At the same time, solid dispersion technology, a classical strategy to increase dissolution, has also been successfully applied to Tanshinone IIA. For example, loading Tanshinone IIA into porous silica carriers (e.g., SYLOID) converts it into an amorphous state, markedly enhancing its water solubility, peak concentration (C_max), area under the curve (AUC), and half-life, ultimately achieving improved oral bioavailability [26].

In lipid-based nano-delivery systems, liposomes are a preferred platform for Tanshinone IIA due to their excellent biocompatibility and modifiable membrane characteristics. One study reported that encapsulating Tanshinone IIA in lipid nanocapsules significantly increased its absorption rate and AUC in vivo (~3.6-fold greater than the unencapsulated drug, *p* ≤ 0.01) and extended its plasma t_1/2 and mean residence time, indicating a favorable long-circulation profile [27]. Additionally, a chitosan/alginate ion-crosslinked Tanshinone IIA nanoparticle formulation improved the drug’s encapsulation efficiency, stability, and mucoadhesion in the gastrointestinal tract, thereby enhancing its absorption under digestive conditions. Nanocrystal liposomes have also been shown to slow Tanshinone IIA clearance and extend its retention time in vivo, achieving sustained drug release.

Furthermore, the advantages of solid dispersion technology in improving Tanshinone IIA dissolution are noteworthy. Differential scanning calorimetry (DSC) and X-ray powder diffraction (XRPD) analyses confirm that Tanshinone IIA remains in an amorphous state within solid dispersions—a physical change crucial for enhancing water solubility and systemic bioavailability. To achieve precise targeting and controlled release, advanced delivery systems such as PEGylated liposomes and polymeric self-assembled nanostructures have also been developed. These systems not only exhibit excellent tissue selectivity and drug accumulation capacity, but also effectively evade first-pass metabolism, thereby increasing the concentration of active drug at target sites [28,29].

In summary, a variety of delivery approaches and formulation innovations have substantially optimized Tanshinone IIA’s in vivo pharmacokinetic behavior and biological effects across different experimental models, providing a solid technical foundation for its clinical translation. Combined with the unique advantages of natural products in regulating bone metabolism and treating osteoporosis, these advances are expected to catalyze more in-depth exploration of Tanshinone IIA’s applications in bone-related diseases.

### 2.3. Pharmacokinetics of Tanshinone IIA (Absorption, Distribution, Metabolism, and Excretion)

Following oral administration, Tanshinone IIA exhibits low bioavailability; its gastrointestinal absorption is chiefly constrained by poor solubility and permeability, and it is particularly unfavorable in acidic media. The compound shows high plasma protein binding (~96–98%), which further limits the unbound fraction and thus effective systemic exposure. To improve absorption, nano-formulations such as nanoparticles and liposomes have been developed with encouraging progress [30]. With respect to distribution, Tanshinone IIA rapidly partitions into the heart, liver, kidneys, and lungs, with the highest levels in the heart and liver, consistent with its principal pharmacological sites of action; it can also cross the blood–brain barrier, suggesting potential neuroprotective activity. Its tissue distribution is time-dependent, showing declining concentrations over time, and organ selectivity varies across tissues, likely reflecting local metabolic features and target engagement [31]. Metabolism occurs predominantly in the liver and involves cytochrome P450 isoforms—particularly CYP3A4, CYP2C9, and CYP1A2—which generate multiple metabolites via hydroxylation, demethylation, and related transformations. Interaction with CYP3A4 may modify its metabolic rate; accordingly, caution is warranted regarding potential drug–drug interactions during co-therapy [32]. Beyond CYP-mediated biotransformation, UDP-glucuronosyltransferases (UGTs) catalyze glucuronidation to form conjugates cleared renally, and reduction reactions can yield dehydrogenated metabolites [33]. Elimination occurs mainly via urine and feces, with a portion excreted in bile; most analytes are present as glucuronide conjugates and oxidative metabolites, whereas only trace amounts of the parent drug are recovered in urine. The elimination half-life is relatively short (~1.5–3 h), limiting maintenance of effective systemic concentrations. Therefore, dose optimization and formulation advancements are critical to enhance its clinical utility [34]. In sum, Tanshinone IIA displays a pharmacokinetic profile characterized by limited absorption, broad tissue distribution, multi-pathway metabolism, and rapid clearance—features that explain current clinical constraints while informing subsequent formulation optimization and dosing strategies.

## 3. Mechanisms of Tanshinone IIA in Bone Tissue Repair

**Figure 2:**
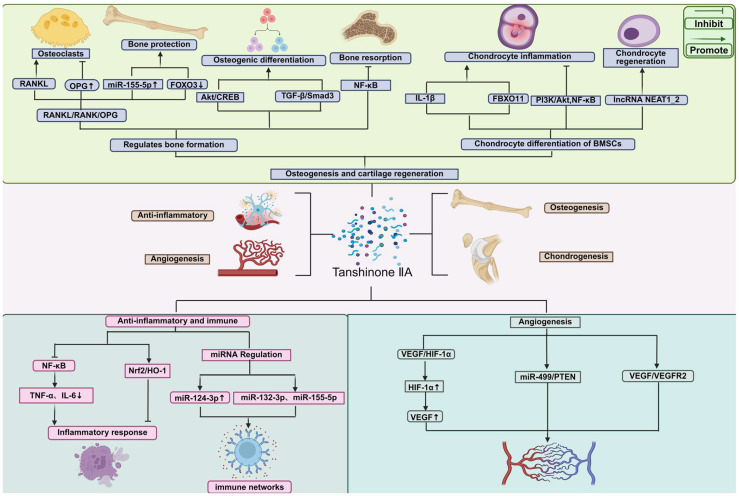
Mechanistic overview of Tanshinone IIA in bone repair, inflammation modulation, and angiogenesis.

As shown in Figure 2, Tanshinone IIA exerts multifaceted effects on bone and cartilage regeneration through the regulation of osteogenesis, osteoclastogenesis, and chondrogenesis. Tanshinone IIA promotes osteoblast differentiation via pathways such as Akt/CREB and TGF-β/Smad3, and inhibits osteoclast-mediated bone resorption by modulating the RANKL/RANK/OPG axis and suppressing NF-κB signaling. It also alleviates chondrocyte inflammation and facilitates chondrogenic differentiation of BMSCs by influencing the PI3K/Akt/NF-κB and NEAT1_2 signaling pathways. Furthermore, Tanshinone IIA exhibits potent anti-inflammatory and immunomodulatory activities by inhibiting NF-κB and activating Nrf2/HO-1 signaling, as well as regulating miRNAs such as miR-124-3p, miR-132-3p, and miR-155-5p. Additionally, Tanshinone IIA promotes angiogenesis by upregulating VEGF and HIF-1α expression and modulating the miR-499/PTEN and VEGF/VEGFR2 pathways. Together, these mechanisms underline the therapeutic potential of Tanshinone IIA in bone metabolic disorders and tissue regeneration.

### 3.1. Osteogenesis-Promoting Effects and Signaling Pathway

Receptor activator of NF-κB ligand (*RANKL*) is a key signaling molecule driving osteoclast differentiation; it induces the expression of multiple critical genes, particularly the transcription factors c-Fos and nuclear factor of activated T-cells cytoplasmic 1 (NFATc1). *RANKL* also activates numerous bone resorption-related enzymes (such as cathepsin K, tartrate-resistant acid phosphatase (TRAP), and various matrix metalloproteinases), thereby accelerating bone tissue breakdown. As shown in Figure 2, studies have demonstrated that Tanshinone IIA significantly inhibits the expression of these *RANKL*-activated molecules, effectively blocking osteoclast formation and function. In regulating bone metabolic balance, Tanshinone IIA also acts through microRNA-mediated mechanisms: it upregulates miR-155-5p and promotes the production of osteoprotegerin (*OPG*), while downregulating *RANKL* and forkhead box O3 (*FOXO3*), thereby favorably modulating bone metabolism. Consequently, Tanshinone IIA exhibits anti-osteolytic effects in both mouse and human osteoblast models, helping delay osteoblast apoptosis and inhibit osteoclast differentiation. In a co-culture system of calvarial osteoblasts and bone marrow cells under lipopolysaccharide (LPS) stimulation, Tanshinone IIA suppressed osteoclastogenesis by adjusting the *RANKL/OPG* expression ratio in osteoblasts. At the same time, it interfered with actin ring formation in the osteoclast cytoskeleton and inhibited NF-κB activation along with the expression of associated factors, further reinforcing its dual inhibitory effect on osteoclast differentiation and bone resorptive activity [35,36,37,38,39].

As shown in Table 1, further research shows that Tanshinone IIA may also intervene in the development of avascular necrosis of the femoral head (ANFH) by targeting bone marrow mesenchymal stem cell (BMSC) function. Under hypoxic conditions, BMSCs can differentiate into both osteogenic and adipogenic lineages. In one study, BMSCs were subjected to osteogenic or adipogenic induction under hypoxia and treated with or without Tanshinone IIA. Alkaline phosphatase (ALP) activity assays, Alizarin Red staining, and qPCR analysis of osteogenic genes (*RUNX2, ALP, COL1A1*) were used to evaluate osteogenesis, while Oil Red O staining and adipogenic marker gene expression were used to assess adipogenesis. The results showed that under hypoxia (Figure 2), Tanshinone IIA significantly increased ALP activity, promoted mineralized nodule formation, and upregulated the expression of osteogenic marker genes. Under adipogenic induction, Tanshinone IIA markedly reduced lipid droplet formation and downregulated adipogenesis-related gene expression. RNA sequencing (RNA-seq) combined with pharmacological inhibitor interventions revealed that Tanshinone IIA enhances the activity of the Akt and TGF-β signaling pathways. Western blot further confirmed that Tanshinone IIA activates the Akt/CREB and TGF-β/Smad3 pathways. Functional blockade experiments found that an Akt inhibitor (Akti1/2) significantly attenuated Tanshinone IIA’s osteogenesis-promoting effect, while a TGF-β receptor inhibitor (SB431542) weakened its adipogenesis-suppressing effect. These findings suggest that Tanshinone IIA synergistically regulates the multi-lineage differentiation fate of BMSCs by concurrently activating both Akt and TGF-β signaling pathways [40].

Studies have confirmed that Tanshinone IIA exerts significant bone-protective effects in both normal bone metabolism and bone loss animal models, although research specifically in the context of bone repair remains limited. Most in vitro studies have concentrated on its inhibition of osteoclast formation, whereas the mechanisms by which it influences BMSC osteogenic differentiation and osteoblast survival are less explored. Some experiments indicate that Tanshinone IIA at 1 μM and 5 μM can promote the early and late stages of BMSC osteogenesis, respectively, whereas a higher dose (20 μM) may exert some inhibitory effect on osteogenesis. As shown in Table 1, another report showed that Tanshinone IIA can reverse MC3T3-E1 osteoblast apoptosis induced by dexamethasone (DEX) or hydrogen peroxide (H_2O_2), an effect possibly related to its inhibition of NADPH oxidase 4 (Nox4)-mediated ROS production and suppression of NF-κB activity. Under oxidative stress conditions, Tanshinone IIA can activate the nuclear factor erythroid 2–related factor 2 (Nrf2) pathway and upregulate multiple antioxidant enzymes, thereby enhancing cellular antioxidant capacity, inhibiting apoptosis, and promoting osteogenic differentiation. In vivo studies have also confirmed that local application of Tanshinone IIA accelerates fracture healing in ovariectomized mice [41]. In summary, Tanshinone IIA possesses multifaceted bone-protective properties: it effectively promotes osteogenesis and inhibits bone resorption while maintaining the dynamic homeostasis of bone metabolism. Therefore, Tanshinone IIA presents a highly promising therapeutic option for treating glucocorticoid-induced osteoporosis (GIOP) and related complications (such as glucocorticoid-induced osteonecrosis of the femoral head, GINFH) [42].

### 3.2. Cartilage Protection and Regeneration

Tanshinone IIA also exhibits broad anti-inflammatory activity. It can suppress the production of inflammatory mediators in various cell types (e.g., cardiomyocytes, macrophages) and reduce inflammatory injury in arthritis models. Notably, as shown in Figure 2, one study reported that Tanshinone IIA can alleviate interleukin-1β (IL-1β)–induced chondrocyte inflammation and extracellular matrix degradation, suggesting that Tanshinone IIA could be a novel therapeutic agent for arthritis. Specifically, IL-1β stimulation of human chondrocytes (CHON-001 cell line) triggers an inflammatory response and cell apoptosis, whereas Tanshinone IIA, by downregulating the ubiquitin ligase FBXO11, concurrently inhibits the PI3K/Akt and NF-κB signaling pathways, significantly mitigating these pathological processes at the molecular level [43].

Furthermore, during inflammatory cytokine–mediated chondrocyte dedifferentiation and stress, the long non-coding RNA *NEAT1_2* plays a critical regulatory role. Under IL-1β stimulation, *NEAT1_2* expression is significantly downregulated, accompanied by suppression of cartilage phenotype–maintaining factors (such as *SOX9* and aggrecan (*ACAN*)), an imbalanced *COL2A1*/*COL1A1* expression ratio, and upregulation of matrix metalloproteinases (MMPs)—all indicating disruption of extracellular matrix (ECM) homeostasis. Importantly, Tanshinone IIA effectively reverses this degenerative phenotypic shift in chondrocytes. As a plant-derived compound capable of modulating lncRNA expression, Tanshinone IIA has garnered attention in this context. Silencing *NEAT1_2* with siRNA confirmed that Tanshinone IIA’s maintenance of the chondrocyte phenotype partly depends on its positive regulation of *NEAT1_2* [44].

In addition, Tanshinone IIA can attenuate the imbalance between ECM synthesis and degradation induced by IL-1β and TNF-α, significantly inhibit chondrocyte apoptosis and oxidative stress, and thereby enhance cell function and promote cartilage tissue regeneration. In a monosodium iodoacetate (MIA)–induced osteoarthritis mouse model, Tanshinone IIA effectively improved subchondral bone remodeling and microarchitecture, and significantly suppressed pathological angiogenesis. Further in vitro research showed that Tanshinone IIA inhibits the angiogenic capacity of primary CD31^hi/Emcn^hi endothelial cells. The underlying mechanism is that Tanshinone IIA reduces the proportion of hypertrophic chondrocytes, decreases the secretion of vascular endothelial growth factor A (VEGFA), and inhibits the activation of VEGF receptor 2 (VEGFR2) and its downstream MAPK signaling pathway, thereby exerting an anti-angiogenic effect [45].

Additional studies demonstrate that Tanshinone IIA also has a remarkable effect in delaying chondrocyte senescence. Following Tanshinone IIA treatment, the expression levels of chondrocyte senescence-associated markers (such as p21, IL-1β, IL-6, and *Mmp3*) are markedly reduced. In aged male mice, Tanshinone IIA suppressed the expression of *Col10a1*, *Mmp13*, *Adamts5*, and *Runx2*, while enhancing *Col2a1* expression, suggesting that it can inhibit hypertrophic changes in chondrocytes. Immunohistochemistry showed that Tanshinone IIA significantly decreased the protein level of cellular communication network factor 1 (CCN1), thereby co-suppressing *Col10a1* and *Mmp13* expression; the mRNA levels mirrored these protein changes. Tanshinone IIA also lowered the cartilage lesion score (OARSI score), preserving the structural integrity of cartilage tissue. CCN1 is known to promote ROS production by binding to integrin receptors and activating the p38/p16^INK4a or p21 pathways, accelerating the progression of osteoarthritis (OA). This study indicates that Tanshinone IIA effectively inhibits CCN1 and the oxidative stress it mediates, thereby slowing the onset and progression of OA [46,47].

In summary, by concurrently targeting multiple pathogenic factors—oxidative stress, apoptosis, matrix metabolism, pathological angiogenesis, and chondrocyte senescence—Tanshinone IIA offers a promising therapeutic approach for cartilage injury repair and degenerative diseases such as osteoarthritis.

### 3.3. Anti-Inflammatory and Immunomodulatory Mechanisms

Tanshinone IIA has demonstrated notable neuroprotective effects in an Alzheimer’s disease model (*APP*^swe/*PS1*^dE9 double-transgenic mice) and in human neuroblastoma SH-SY5Y cells, effectively alleviating endoplasmic reticulum stress–induced neuronal apoptosis. Meanwhile, numerous in vivo and in vitro studies show that Tanshinone IIA possesses broad-spectrum anti-inflammatory capabilities, significantly improving pathological features of neurodegenerative diseases—including synaptic and neuronal damage, astroglial activation, abnormally elevated inflammatory cytokines, and Aβ deposition—accompanied by restoration of spatial learning and cognitive function. Mechanistically, as shown in Figure 2, Tanshinone IIA inhibits activation of the receptor for advanced glycation end products (RAGE)/NF-κB pathway, reducing the production of pro-inflammatory cytokines (TNF-α, IL-6, IL-1β) in the brains of *APP/PS1* mice as well as in BV2 microglia and U87 astrocytes [48].

Further studies emphasize that the Nrf2/HO-1 pathway plays a central role in maintaining immune cell redox homeostasis and suppressing the amplification of inflammatory signaling, and that loss of Nrf2 exacerbates systemic inflammatory responses in sepsis models. Tanshinone IIA can block lipopolysaccharide (LPS)-induced TLR4/NF-κB signaling, significantly downregulating the expression of key inflammatory mediators in RAW264.7 macrophages, including inducible nitric oxide synthase (iNOS), cyclooxygenase-2 (COX-2), IL-1β, and IL-6. Notably, COX-2 is the rate-limiting enzyme converting arachidonic acid to, and nitric oxide produced by iNOS is an important signaling molecule in prostaglandins inflammation. Tanshinone IIA’s inhibition of these enzymes helps alleviate Aβ-induced neurotoxicity, further underscoring its potential in modulating neuroinflammation [49].

NF-κB is a central transcription factor for pro-inflammatory genes that, under pathological stimuli and oxidative stress, can become persistently activated, driving the expression of inflammatory factors such as TNF-α, IL-6, IL-1β, and IL-8, and influencing cell proliferation and apoptosis. Experiments have shown that Tanshinone IIA effectively suppresses NF-κB activation in various cell types (e.g., murine mammary epithelial cells [mMECs], human monocytes [THP-1], murine macrophages [RAW264.7]), thereby significantly downregulating the levels of downstream inflammatory cytokines [50].

As shown in Figure 2, in a rheumatoid arthritis fibroblast-like synoviocyte (FLS) model, Tanshinone IIA inhibited the TNF-α–mediated inflammatory cascade, alleviating inflammatory symptoms in adjuvant-induced arthritis mice. Notably, Tanshinone IIA also exerts immunomodulatory effects by regulating microRNA networks: it downregulates multiple pro-inflammatory chemokines (e.g., IL-8, *CXCL1*/GRO-α, *CCL2*/MCP-1, *CCL5*/RANTES) while upregulating miR-124-3p, and it suppresses the TNF-α-induced expression of miR-132-3p and miR-155-5p. These findings indicate that Tanshinone IIA can inhibit the propagation of inflammation through fine-tuned, miRNA-mediated regulation [51].

More in-depth mechanistic studies have revealed that in LPS-stimulated mouse bone marrow–derived macrophages (BMDMs), Tanshinone IIA reduces succinate accumulation by inhibiting succinate dehydrogenase (SDH) activity. This metabolic shift lowers hypoxia-inducible factor-1α (HIF-1α) levels, suppresses the production of IL-1β and IL-6, and promotes the release of anti-inflammatory cytokines such as IL-1 receptor antagonist (IL-1RA) and IL-10. Additionally, Tanshinone IIA raises the intracellular NAD^+^/NADH ratio, which maintains the activity of sirtuin 2 (Sirt2); active Sirt2 binds to α-tubulin and prevents its acetylation, thereby inhibiting assembly of the NLRP3 inflammasome. Knocking down Sirt2 or using its specific inhibitor AGK-2 significantly diminished Tanshinone IIA’s anti-inflammatory effects, suggesting that this mechanism is Sirt2-dependent. In an LPS-induced acute inflammation mouse model, pretreatment with Tanshinone IIA effectively reduced systemic inflammation, evidenced by increased serum IL-13 and decreased IL-10 levels, along with corresponding metabolic reprogramming of macrophages. These findings further validate Tanshinone IIA’s key role in immunometabolic regulation [52].

### 3.4. Angiogenic Effects

Vascular endothelial growth factor (VEGF) is a key pro-angiogenic factor that regulates neovascularization, playing a critical role particularly in post–myocardial infarction (MI) angiogenesis. As shown in Figure 2, under hypoxic conditions, hypoxia-inducible factor-1α (HIF-1α) promotes VEGF expression through transcriptional regulation, thereby activating angiogenesis-related pathways and mitigating pathological ventricular remodeling. HIF-1α is widely considered the primary transcription factor controlling VEGF transcription in ischemic microenvironments. Studies have shown that Tanshinone IIA can effectively increase the expression of HIF-1α and VEGF, thereby promoting angiogenesis and significantly improving left ventricular remodeling after MI [53].

In one study, histological (H&E) staining and immunohistochemistry showed that psychologically stressed mice exhibited significantly reduced myocardial capillary density and CD31 expression in cardiac tissue. However, after intervention with either a selective glucocorticoid receptor antagonist (CORT125134) or Tanshinone IIA, these indices improved markedly. Concurrently, CD31, VEGF, and angiopoietin-2 (ANG2) expression levels were correspondingly increased. This suggests that psychological stress may suppress endometrial angiogenesis by activating the glucocorticoid receptor (GC/GR) pathway, whereas Tanshinone IIA may counteract this effect and promote new blood vessel formation in the endometrium by inhibiting excessive GC/GR pathway activation, thereby increasing embryo implantation rates. Notably, a 30 mg/kg dose of Tanshinone IIA produced a pro-angiogenic effect comparable to that of aspirin, underscoring its therapeutic potential as an angiogenic agent. Additionally, the study confirmed that Tanshinone IIA ameliorated the adverse impact of psychological stress on embryo implantation by improving mitochondrial function, reducing cellular oxidative stress, and promoting endometrial neovascularization via modulation of the GC/GR pathway [54].

Further research has revealed that Tanshinone IIA also actively improves cardiac function after MI, in part through its regulation of angiogenesis. Based on miRNA microarray and bioinformatics analysis, as shown in Figure 2, Tanshinone IIA treatment was found to significantly reduce the expression of miR-499 in a mouse myocardial infarction model, while its target gene *PTEN* was markedly upregulated. Functional experiments confirmed that inhibiting miR-499 significantly promotes human umbilical vein endothelial cell (HUVEC) proliferation, migration, and tube formation. A dual-luciferase reporter assay further verified that *PTEN* is a direct target of miR-499-5p. Therefore, Tanshinone IIA can induce therapeutic angiogenesis by modulating the miR-499-5p/*PTEN* axis, effectively promoting the recovery of cardiac function after MI [55].

Studies in the central nervous system have shown that Tanshinone IIA can enhance the protein expression of VEGF and its receptor VEGFR2 in ischemic brain tissue, suggesting that its mechanism of action may involve activating the VEGF/VEGFR2 pathway to promote angiogenesis in the brain. Follow-up experiments using ELISA quantified VEGF levels in brain homogenates and serum. In an ischemic stroke model, compared with the sham group, cerebral ischemia caused a decrease in mRNA levels of multiple pro-angiogenic factors (VEGF-A, Ang-1, VEGFR-2, CD31, and basic FGF). After Tanshinone IIA intervention, these indicators were all significantly upregulated, indicating that Tanshinone IIA can restore multiple angiogenic signaling pathways. In contrast, edaravone treatment only slightly increased Ang-1 expression and had no obvious effect on the other angiogenic markers, suggesting that Tanshinone IIA exhibits a more pronounced therapeutic advantage in regulating angiogenesis after ischemic stroke [56].

Another study focused on Tanshinone IIA’s potential role in modulating pathological vascular remodeling. In vivo, Tanshinone IIA significantly inhibited neointima formation after arterial injury. In vitro, Tanshinone IIA suppressed the excessive proliferation of rat vascular smooth muscle cells (VSMCs) induced by platelet-derived growth factor-BB (PDGF-BB) and, in the presence of rapamycin, promoted the expression of smooth muscle differentiation marker genes. Meanwhile, Tanshinone IIA treatment markedly upregulated the transcription factor KLF4, and KLF4 overexpression further enhanced the inhibitory effect of Tanshinone IIA on VSMC proliferation. Taken together, these results suggest that Tanshinone IIA may effectively participate in and regulate pathological vascular remodeling by modulating KLF4-mediated VSMC phenotypic switching, highlighting its broad prospects as a candidate drug for vascular diseases [57].

## 4. Systemic Effects of Tanshinone IIA

**Figure 3:**
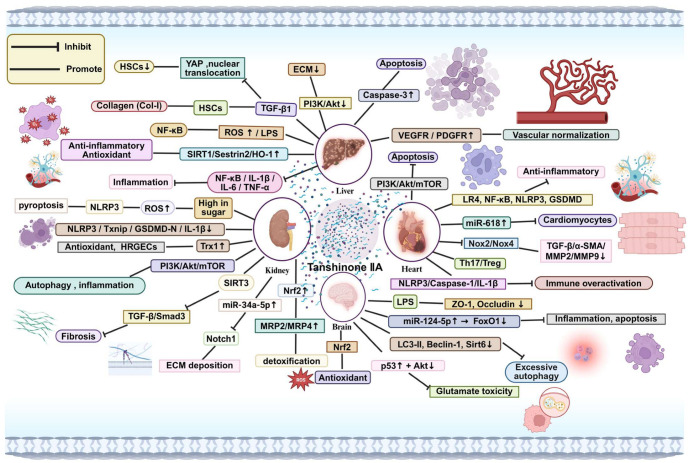
Mechanistic pathways of Tanshinone IIA (T-IIA) in multiorgan-protection.

As shown in Figure 3, this diagram illustrates the multifaceted therapeutic effects of Tanshinone IIA in various organs. Tanshinone IIA exerts anti-inflammatory, antioxidant, and anti-fibrotic actions through modulation of key signaling pathways, including NF-κB, PI3K/Akt/mTOR, and SIRT3, and by regulating miRNA expression (e.g., miR-618, miR-34a-5p). In the liver, Tanshinone IIA mitigates inflammation and fibrosis by inhibiting ROS production and modulating the NF-κB pathway, thereby enhancing liver detoxification and reducing fibrosis markers. In the kidney, Tanshinone IIA ameliorates oxidative stress, inflammation, and fibrosis through PI3K/Akt signaling, while promoting antioxidative effects via Nrf2 activation. In the heart, Tanshinone IIA normalizes vascular function, reduces apoptosis in cardiomyocytes, and inhibits excessive immune activation by modulating the Th17/Treg balance. In the brain, Tanshinone IIA exerts neuroprotective effects through antioxidation and modulation of autophagy, preventing glutamate-induced toxicity and inflammation. Furthermore, Tanshinone IIA promotes vascular normalization by upregulating VEGFR and PDGFR expression, while regulating extracellular matrix (ECM) deposition and fibrosis via TGF-β signaling. These findings suggest Tanshinone IIA’s broad potential for therapeutic interventions in inflammation-driven diseases and organ damage.

### 4.1. Effects on the Liver

The liver is a central organ for maintaining internal homeostasis, responsible for metabolic regulation, detoxification, and other physiological functions. Due to its continuous involvement in nutrient metabolism and xenobiotic detoxification, the liver is highly sensitive to various endogenous and exogenous harmful factors, making it prone to functional disorders and tissue injury. Environmental pollutants, drug metabolites, excessive alcohol consumption, and pathogenic infections have all been shown to cause liver damage to varying degrees. The pathological progression of liver injury involves the coordinated action of multiple signaling pathways and cell types and is usually accompanied by widespread programmed hepatocyte death. Activation of oxidative stress and immune-mediated inflammation is considered a key event in the onset and progression of liver injury. As one of the most metabolically active organs, the liver is especially vulnerable to oxidative damage. Numerous studies have confirmed that disruption of hepatic redox balance is not only a trigger of inflammatory responses and metabolic disturbances, but is also closely associated with various proliferative liver diseases. Reactive oxygen species (ROS), primarily generated by mitochondrial and ER cytochrome P450 systems, can accumulate excessively; such ROS buildup leads to lipid peroxidation, protein conformational changes, and nucleic acid damage, resulting in declining liver function and structural tissue damage [58].

Tanshinone IIA, as the major lipophilic active component of Danshen, has been extensively studied and shown to have significant hepatoprotective effects. As shown in Figure 3, studies indicate that Tanshinone IIA effectively reduces serum levels of multiple enzymes indicative of hepatocyte injury, such as aspartate aminotransferase (AST), alanine aminotransferase (ALT), lactate dehydrogenase (LDH), and γ-glutamyl transferase (γ-GT). Histopathological examinations, clinical symptom evaluations, and hydroxyproline (Hyp) content measurements further demonstrated that Tanshinone IIA significantly delays the progression of liver fibrosis. Network pharmacology analysis and KEGG pathway enrichment suggested that the anti-fibrotic mechanism of Tanshinone IIA may be closely related to its regulation of the PI3K/Akt signaling pathway, providing a theoretical basis for further elucidating its molecular mechanism [59].

Additionally, Tanshinone IIA exhibits notable lipid-regulating effects, effectively lowering plasma lipid levels and inhibiting the formation of atherosclerotic plaques at the aortic root. At the same time, Tanshinone IIA significantly reduces total cholesterol (TC), triglycerides (TG), and lipid droplet accumulation in the liver. Given the liver’s central role in cholesterol synthesis, transport, and metabolism, hepatic dysfunction is closely linked to the development of atherosclerosis. Notably, elevated ALT levels have been identified as one of the early predictors of cardiovascular risk. In one study, Tanshinone IIA markedly improved liver histopathology and reduced AST and ALT levels, suggesting that by modulating hepatic lipid metabolic networks, it exerts a potential anti-atherosclerotic effect [60].

In liver fibrosis models, as shown in Figure 3, Tanshinone IIA was found to inhibit transforming growth factor-β (TGF-β)–mediated hepatic stellate cell (HSC) activation and excessive type I collagen (Col-I) synthesis. Mechanistic studies indicate that yes-associated protein (YAP) is persistently overexpressed during liver fibrosis, promoting fibroblast-like transdifferentiation, and YAP upregulation is regarded as a hallmark of fibrosis progression. In this study, Tanshinone IIA effectively suppressed HSC proliferation and collagen deposition by inhibiting the upregulation and nuclear translocation of YAP, thereby exerting an anti-fibrotic effect [61].

In hepatocellular carcinoma (HCC) studies, Tanshinone IIA has shown significant anti-tumor vascular remodeling effects. Research indicates that Tanshinone IIA induces apoptosis of tumor cells by modulating the expression of caspase-3 and CD31. Moreover, when used in combination with resveratrol, Tanshinone IIA further enhanced cell cycle arrest and apoptosis, causing tumor cells to stall in the G1 phase. In addition, Tanshinone IIA can promote vessel normalization and improve the hypoxic tumor microenvironment by upregulating the expression of VEGF receptors and PDGF receptors, thereby reducing the risk of postoperative metastasis of liver cancer cells [62].

In a sepsis-induced liver injury model, Tanshinone IIA displayed significant protective effects on liver function. Tanshinone IIA markedly improved the pathological changes in mouse liver tissue caused by cecal ligation and puncture (CLP) and increased liver function scores. Mechanistic analysis showed that Tanshinone IIA activates the SIRT1/Sestrin2/HO-1 signaling pathway, significantly upregulating these proteins and inhibiting the release of pro-inflammatory cytokines. When pharmacological inhibitors or siRNA were used to interfere with SIRT1 or Sestrin2 expression, Tanshinone IIA’s protective effect was clearly diminished, suggesting that Sestrin2 acts as a key downstream mediator of SIRT1 in the regulation of immune homeostasis. By activating the SIRT1/Sestrin2/HO-1 axis, Tanshinone IIA counteracts LPS-induced inflammatory responses, demonstrating promising therapeutic potential in ameliorating sepsis-related hepatic dysfunction [63].

### 4.2. Effects on the Kidney

Diabetic nephropathy (DN) is one of the most common and destructive chronic complications in diabetic patients, typically presenting early on as microalbuminuria, which is considered a warning sign of renal impairment. As the disease progresses, patients develop classic pathological changes such as glomerular hypertrophy, mesangial matrix expansion, glomerulosclerosis, and renal interstitial fibrosis. Danshen has been an important traditional medicine for treating DN, and its main active component, Tanshinone IIA, has demonstrated consistent renoprotective efficacy in multiple in vivo and in vitro models. Studies have confirmed that Tanshinone IIA can reduce kidney tissue damage in db/db diabetic mice, improve key urinary and serum biochemical indices, and decrease the expression levels of the NLRP3 inflammasome and its upstream regulator, Txnip. In high glucose–induced human renal glomerular endothelial cells (HRGECs), Tanshinone IIA effectively countered ROS accumulation, prevented the increase in propidium iodide–positive (necrotic) cells, and preserved cell viability. It also inhibited the expression of cleaved gasdermin D (GSDMD-N), activated caspase-1, IL-1β, NLRP3, and Txnip, while restoring thioredoxin-1 (Trx1) levels, thereby reducing pyroptosis and inflammatory injury to the cells [64].

Further experimental evidence, as shown in Figure 3, indicates that Tanshinone IIA significantly improves DN-induced renal structural abnormalities and dysfunction. In a podocyte model, Tanshinone IIA upregulated key podocyte structural proteins (synaptopodin, podocin) and autophagy markers (LC3-II/I and Beclin-1) (*p* < 0.05), while downregulating the autophagy substrate p62, the macrophage marker F4/80, NF-κB p65, and inflammatory factors IL-1β, TNF-α, and IL-6 (*p* < 0.05). This suggests that Tanshinone IIA alleviates podocyte injury by activating autophagy pathways and suppressing immune-inflammatory responses. Simultaneously, Tanshinone IIA significantly inhibited the phosphorylation of the PI3K/Akt/mTOR signaling pathway, further supporting that it exerts renal protection by balancing autophagic and inflammatory signaling through this pathway [65].

Doxorubicin (DOX) is a broad-spectrum antitumor drug, but its pronounced nephrotoxicity and associated renal interstitial fibrosis severely limit its clinical use. In a drug-induced kidney injury model, mice were given intraperitoneal injections of DOX (3 mg/kg every three days for 7 doses, cumulative 21 mg/kg), followed by administration of Tanshinone IIA (5 or 10 mg/kg/day) for 28 days. Tanshinone IIA treatment significantly reduced DOX-induced blood urea nitrogen (BUN) levels, increased SOD activity, decreased ROS production, and improved renal tubular epithelial cell proliferation and mitochondrial integrity. At the same time, Tanshinone IIA significantly reduced collagen deposition in renal tissue, increased ATP production and the activity of mitochondrial complex I, downregulated TGF-β1 and phosphorylated Smad3 (p-Smad3) levels, and enhanced the expression of the mitochondrial functional protein SIRT3. RNA interference experiments further confirmed that knocking down SIRT3 attenuated Tanshinone IIA’s inhibitory effects on TGF-β1 and p-Smad3, suggesting that its anti-fibrotic mechanism is achieved through SIRT3-mediated regulation of the TGF-β pathway [66].

Tanshinone IIA similarly has a beneficial effect on diabetes-related renal fibrosis. Studies found that, as shown in Figure 3, miR-34a-5p expression is significantly downregulated in a DN model, and Tanshinone IIA intervention restores its level. Further experiments showed that silencing miR-34a-5p weakens Tanshinone IIA’s anti-fibrotic effects, suggesting that miR-34a-5p is an important mediator of Tanshinone IIA’s action. A dual-luciferase reporter assay confirmed that miR-34a-5p can directly target Notch1 and suppress its expression. Therefore, it is speculated that Tanshinone IIA upregulates miR-34a-5p and, via the miR-34a-5p/Notch1 axis, modulates downstream signaling to slow or reverse the progression of renal fibrosis [67]. Additionally, Tanshinone IIA provides clear protective effects against acute nephrotoxicity induced by acetaminophen (APAP). In animal experiments, Tanshinone IIA reduced serum creatinine levels and ameliorated histological damage to renal tubules. The protective mechanism is thought to be related to enhancing the clearance of the toxic metabolite N-acetyl-p-benzoquinone imine (NAPQI). Studies noted that in *Nrf2*^+/+^ mice, Tanshinone IIA significantly upregulated Nrf2 and its downstream targets Mrp2 and Mrp4 at both mRNA and protein levels, whereas this effect disappeared in *Nrf2*^−/−^ mice. In vitro, Tanshinone IIA similarly increased nuclear accumulation of Nrf2 in HK-2 renal cells, thereby activating the Nrf2–MRP2/4 pathway to boost cellular detoxification and cytoprotection, providing an effective strategy for preventing APAP-related kidney injury [68].

Further research found that Tanshinone IIA can exert nephroprotective effects via G protein–coupled estrogen receptor (GPER)-mediated signaling. GPER plays a central role in regulating glucose/lipid metabolism, oxidative stress, and apoptosis. In a cold storage–induced renal injury model, Tanshinone IIA promoted the phosphorylation of MEK, ERK1/2, and GSK-3β, activating the MEK/ERK1/2/GSK-3β axis and thereby reducing tissue damage. When a GPER-specific antagonist (G15) was used, Tanshinone IIA’s effects on pathway activation and anti-apoptosis were significantly weakened, demonstrating that it exerts renal protection via a GPER-dependent mechanism. In summary, Tanshinone IIA modulates the GPER–MEK/ERK1/2/GSK-3β axis to regulate cell survival and stress responses, effectively alleviating cold storage–related renal injury [69].

### 4.3. Effects on the Heart

Tanshinone IIA has been widely used to prevent tissue damage and organ failure caused by cardiovascular events such as myocardial infarction and arrhythmias. Tanshinone IIA possesses significant antioxidant and antithrombotic effects and can improve vascular smooth muscle cell (VSMC) function, thereby exerting beneficial effects in acute myocardial infarction (AMI). In addition, Tanshinone IIA shows a clear cardioprotective effect in myocardial ischemia–reperfusion injury (IRI), and its mechanism is closely related to the PI3K/Akt/mTOR signaling pathway. One study found that in a myocardial IRI model, pre-administration of Tanshinone IIA significantly reduced myocardial infarct size and lowered cardiomyocyte apoptosis rates [70]. As shown in Figure 3, Tanshinone IIA treatment markedly improved cardiac function, attenuated histopathological damage to myocardial tissue, decreased serum levels of N-terminal pro–B-type natriuretic peptide (NT-proBNP), IL-1β, and IL-18, and effectively inhibited cardiomyocyte apoptosis. Further studies showed that Tanshinone IIA downregulates the expression of pyroptosis-related proteins in myocardial tissue, including Toll-like receptor 4 (TLR4), NF-κB p65, IL-1β, pro–IL-1β, NLRP3, caspase-1, and GSDMD-N. Moreover, Tanshinone IIA significantly increased the survival of H9c2 cardiomyocytes under hypoxia/reoxygenation, inhibited cell apoptosis, and reduced the levels of pyroptosis-related proteins. Importantly, Tanshinone IIA was found to inhibit the translocation of NF-κB p65 into the nucleus [71].

Another study found that elevated tissue inhibitor of metalloproteinases (TIMP) levels are closely associated with the extent of myocardial fibrosis and structural deterioration, suggesting that low expression of miR-618 may promote cardiac fibrosis. Tanshinone IIA treatment restored miR-618 levels, significantly improving fibrotic progression in mouse cardiac fibroblasts and rat hearts [72]. In a myocardial infarction–induced heart failure model, an imbalance between oxidants and antioxidants was effectively corrected by Tanshinone IIA; by suppressing oxidative stress, Tanshinone IIA slowed the development of cardiac fibrosis during heart failure progression. Further research found that Tanshinone IIA may act as an effective inhibitor of LPS-induced cardiac fibrosis in mice, likely through partial inhibition of NADPH oxidase 2 (Nox2). It was shown that overexpression of *Nox4* could reverse the improvements in cardiac function conferred by Tanshinone IIA in heart-failure rats: *Nox4* overexpression negated Tanshinone IIA’s ability to reduce left ventricular type I and III collagen, TGF-β, α-smooth muscle actin (α-SMA), MMP2, and MMP9. Therefore, *Nox4* plays a key role in Tanshinone IIA’s suppression of heart failure and myocardial fibrosis [73].

Further studies indicate that Tanshinone IIA also has significant efficacy in a myocardial infarction/reperfusion (MI/R) injury rat model. Tanshinone IIA treatment markedly improved cardiac function post-MI/R, as evidenced by increased atrioventricular peak velocity, ejection fraction (EF%), and fractional shortening (FS%), along with a clear reduction in myocardial infarct size. TTC staining demonstrated that Tanshinone IIA treatment significantly reduced myocardial injury, and H&E staining revealed that the Tanshinone IIA–treated group had notably improved myocardial tissue morphology with reduced inflammatory cell infiltration. In addition, Tanshinone IIA significantly dampened the inflammatory response and effectively inhibited NLRP3 inflammasome activation, downregulating NLRP3 and caspase-1 protein levels while also suppressing the mRNA expression of NLRP3, caspase-1, IL-1β, IL-18, and forkhead box P3 (*FoxP3*). Most importantly, Tanshinone IIA was able to modulate the Th17/Treg immune balance in MI/R rats [74].

### 4.4. Effects on the Brain

The blood–brain barrier (BBB) is composed of a highly selective physical barrier and transport systems, maintaining central nervous system homeostasis. Many brain disorders (e.g., autoimmune encephalopathy, Alzheimer’s disease, Huntington’s disease) are closely associated with BBB dysfunction. The integrity of the BBB depends on tight junction complexes and restricted trans-endothelial transport. Research indicates that, as shown in Figure 3, Tanshinone IIA can effectively inhibit the increase in BBB permeability in an LPS-induced brain injury model, confirming its protective effect on the BBB. In this model, LPS significantly downregulated the expression of the tight junction proteins ZO-1 and occludin, whereas Tanshinone IIA treatment restored ZO-1 and claudin-5 levels in LPS-stimulated brain microvascular endothelial cells (bEnd.3). These results suggest that Tanshinone IIA maintains BBB integrity by upregulating tight junction proteins, thereby exerting neuroprotective effects in brain injury [75].

In a middle cerebral artery occlusion/reperfusion (MCAO/R) model, Tanshinone IIA treatment significantly reduced infarct volume, lowered brain water content, and improved neurological deficit scores in rats, with a clear attenuation of cellular damage. Additionally, Tanshinone IIA effectively suppressed the inflammation and neuronal apoptosis induced by ischemia–reperfusion: it decreased the expression of inflammatory cytokines IL-1β, IL-6, and TNF-α; reduced the levels of the pro-apoptotic protein Bax and activated caspase-3; and increased the expression of the anti-apoptotic protein Bcl-2. In an in vitro oxygen-glucose deprivation/reoxygenation (OGD/R) neuronal model, Tanshinone IIA similarly inhibited the expression of inflammatory factors and significantly reduced cell apoptosis. More importantly, Tanshinone IIA promoted the expression of miR-124-5p. Transfection of a miR-124-5p mimic reproduced neuroprotective effects similar to Tanshinone IIA, while transfection of a miR-124-5p inhibitor abrogated Tanshinone IIA’s protective effects. Mechanistic studies revealed that miR-124-5p acts by targeting forkhead box O1 (*FoxO1*); either inhibiting miR-124-5p or overexpressing *FoxO1* weakened Tanshinone IIA’s protection against cerebral I/R injury, and simultaneously inhibiting miR-124-5p while overexpressing *FoxO1* further diminished Tanshinone IIA’s efficacy. Thus, Tanshinone IIA attenuates ischemia–reperfusion brain injury by modulating the miR-124-5p/*FoxO1* axis to suppress neuroinflammation [76].

Numerous studies have shown that Tanshinone IIA confers significant neuroprotection in cerebral ischemic injury. Research over the past decade confirms that Tanshinone IIA can protect against cerebral I/R damage through multiple mechanisms. Tanshinone IIA has been found to reduce the expression of activated caspase-3 in ischemic cortex and increase Bcl-2 protein expression, suggesting that its neuroprotective effect in focal cerebral I/R injury is closely related to the inhibition of apoptosis. Another study noted that Tanshinone IIA exerts neuroprotective effects by activating an Nrf2-dependent antioxidant response. More recent research indicates that Tanshinone IIA reduces the number of inflammatory cells in brain tissue after I/R and significantly decreases the upregulation of autophagy-related proteins (such as LC3-II, Beclin-1, Sirt6) induced by ischemia [77]. Further experimental evidence suggests that Tanshinone IIA’s neuroprotection in cerebral I/R injury may also involve regulating histone acetylation to inhibit excitotoxicity and apoptosis. In a rat MCAO model, systemic Tanshinone IIA treatment significantly lowered neurological deficit scores and infarct volume. In in vitro experiments, an OGD/R model using primary hippocampal neurons demonstrated no significant cytotoxicity from Tanshinone IIA at concentrations below 15.07 μg/mL, confirming its safety in neurons. Overall, these results indicate that Tanshinone IIA has a remarkable neuroprotective effect against cerebral I/R injury [78]. By reducing inflammation, inhibiting apoptosis, and countering oxidative stress, Tanshinone IIA achieves significant neuroprotection.

Studies also show that Tanshinone IIA can counteract ischemic brain injury via additional pathways. Some investigations found that Tanshinone IIA can decrease the expression of autophagy-related proteins Beclin-1 and LC3-II, thereby inhibiting excessive autophagy, which improves neurological deficits and reduces cerebral edema in rats. Ischemic brain injury often involves massive glutamate release and overactivation of glutamate receptors, leading to increased ROS production, ER stress, and inflammasome activation, which in turn cause neuronal damage. One study demonstrated that, as shown in Figure 3, Tanshinone IIA can attenuate glutamate-induced cytotoxicity by inhibiting p53 activation (phosphorylation) and promoting Akt expression, thereby mitigating ischemic brain damage [79].

### 4.5. Effects on the Nervous System

The pathogenesis of neurodegenerative diseases is usually accompanied by pathological processes such as oxidative stress, cell apoptosis, inflammatory responses, and neuronal injury. Tanshinone IIA, as a natural compound with broad pharmacological actions, has notable advantages in suppressing oxidative stress, modulating NF-κB signaling, and enhancing the expression of brain-derived neurotrophic factor (BDNF). Its pharmacological profile indicates that Tanshinone IIA has outstanding neuroprotective effects, capable of effectively addressing multiple neurodegenerative diseases and neuropsychiatric disorders, with its anti-inflammatory properties being especially prominent. Tanshinone IIA’s mechanism of action primarily involves inhibiting the expression of inflammatory mediators (e.g., IL-1β, TNF-α, IL-6), reducing excessive microglial activation, and alleviating neuroinflammation. The presence of the BBB often impedes many drugs from entering the CNS, posing a major challenge for treating neurodegenerative diseases. However, as a small, lipophilic molecule, Tanshinone IIA can effectively cross the BBB, making it a highly promising therapeutic option [80]. Research shows that Tanshinone IIA not only decreases acetylcholinesterase (AChE) activity, elevates acetylcholine (ACh) levels, improves cholinergic neuronal function, and enhances choline acetyltransferase (ChAT) activity, but it also effectively promotes neurogenesis and significantly increases the expression of the synaptic marker PSD-95 (a protein closely related to learning and memory) in an Alzheimer’s disease (AD) mouse model. Further studies indicate that after Tanshinone IIA treatment, neuronal damage in these mice was markedly reduced, and Nissl staining showed a more orderly neuronal arrangement [81].

Moreover, studies have shown that Tanshinone IIA can significantly prevent LPS-induced reductions in cell viability, inhibit the excessive activation of human U87 glioma cells, and decrease mRNA levels of IL-1β, TNF-α, and IL-6. Its neuroprotective effect is mainly achieved via inhibition of the TLR4/NF-κB/MAPK signaling pathway, thereby reducing LPS-induced neurotoxicity and neuroinflammation. In AD models, Tanshinone IIA exhibits multiple pharmacological effects, including anti-Aβ, anti-inflammatory, anti-apoptotic, and antioxidant actions. For example, Tanshinone IIA protects primary cortical neurons from Aβ_25–35-induced neurotoxicity and by preventing the aberrant translocation of cyclin-dependent kinase 5 (CDK5), it significantly reduces levels of phosphorylated tau protein [82].

Another study noted that inducible nitric oxide synthase (*iNOS*), matrix metalloproteinase-2 (*MMP-2*), and NF-κB p65 are closely related to AD progression, suggesting that Tanshinone IIA may lower AD risk by inhibiting the transcription and translation of these genes [83]. It is worth mentioning that the antioxidant and anti-inflammatory effects of Tanshinone IIA have been validated in multiple studies. One study found that Tanshinone IIA significantly alleviated hydrogen peroxide–induced damage to human umbilical vein endothelial cells, demonstrating potent protective effects. Further research showed that Tanshinone IIA’s antioxidant potential reduces Aβ-induced neurotoxicity in cortical neurons and inhibits NF-κB–mediated *iNOS* and *MMP-2* expression, thereby lowering the risk of Aβ-induced neuroinflammation [84].

### 4.6. Effects of Tanshinone IIA on Genetic Material

Extensive preclinical studies have systematically evaluated the effects of Tanshinone IIA on the integrity of genetic material and the associated regulatory pathways. Across multiple tumor cell models, Tanshinone IIA robustly induces apoptosis without clear evidence of genotoxic or mutagenic activity. Studies by Lee et al. demonstrated that Tanshinone IIA exerts pronounced cytotoxicity primarily through induction of apoptosis while maintaining intracellular glutathione/oxidized glutathione (GSH/GSSG) redox homeostasis, indicating that its pro-apoptotic effect is unlikely to be driven by direct DNA mutation or genotoxic stress [85,86].

Mechanistically, Tanshinone IIA can induce DNA fragmentation by perturbing DNA repair processes, thereby triggering programmed cell death. In the pituitary corticotroph adenoma cell line AtT-20, Tanshinone IIA treatment elicited a canonical apoptotic phenotype characterized by DNA fragmentation, as confirmed by TUNEL assay. Further studies show that Tanshinone IIA suppresses expression of the DNA repair enzyme MGMT (O^6-methylguanine-DNA methyltransferase) downstream of Wnt/β-catenin signaling, thereby attenuating DNA repair capacity, facilitating the accumulation of DNA lesions, and promoting apoptosis [87,88].

Taken together, current evidence indicates that Tanshinone IIA does not directly elicit mutagenic genotoxicity; rather, it influences genomic integrity by modulating intracellular signaling pathways. Its principal actions include downregulation of DNA repair enzymes, enhancement of DNA-damage signaling, and induction of DNA fragmentation. These features underscore Tanshinone IIA’s therapeutic potential as an agent that, instead of directly damaging DNA, achieves antitumor efficacy by regulating gene expression and DNA repair mechanisms under pathological conditions.

## 5. Efficacy and Toxicity Evaluation of Tanshinone IIA in Animal Models of Bone Repair

### 5.1. Preclinical Studies on Tanshinone IIA-Promoted Bone Repair in Animal Models

As shown in Table 2, in a rat osteoarthritis (OA) model induced by anterior cruciate ligament transection (ACLT) combined with medial meniscectomy (MMx), Tanshinone IIA was administered orally at 0.25–0.5 mg/kg for 28 days. The model group exhibited obvious degenerative changes in knee articular cartilage, with Mankin scores significantly higher than normal controls. At the 0.5 mg/kg dose, Tanshinone IIA effectively inhibited cartilage matrix degradation, markedly lowered the Mankin score (*p* < 0.002), and reduced inflammatory cell infiltration and structural damage in the synovial tissue. Notably, at this dose, the chondrocyte apoptosis rate on day 29 dropped from 41% in the model group to just 2%. Mechanistically, Tanshinone IIA downregulated the expression of MMP family members in OA cartilage while upregulating tissue inhibitors of metalloproteinases (TIMPs), concurrently reducing the serum concentrations of inflammatory mediators IL-1β, TNF-α, and nitric oxide (NO). At the same time, Tanshinone IIA promoted the expression of bone morphogenetic protein (BMP) and TGF-β in cartilage tissue, suggesting that this compound alleviates chondrocyte apoptosis and cartilage degeneration through multiple synergistic actions [89].

In osteoporosis research, Tanshinone IIA likewise has shown significant efficacy. As shown in Table 2, in ovariectomized (OVX) rats, the model animals exhibited alveolar bone loss, thinner and more widely spaced trabeculae, and decreased proliferation and colony-forming ability of BMSCs, accompanied by reduced stemness and upregulated senescence markers. Tanshinone IIA intervention upregulated the mRNA expression of 3-phosphoglycerate dehydrogenase (PHGDH), thereby delaying BMSC senescence and restoring their stemness, whereas use of a PHGDH inhibitor reversed this effect. OVX surgery also decreased the methylation level of a CpG island in the PHGDH promoter in BMSCs; after Tanshinone IIA treatment, BMSC proliferation and stemness indices returned to levels comparable to sham-operated controls [90].

As shown in Table 2, in an STZ-induced diabetic mouse model, eight weeks of intraperitoneal Tanshinone IIA administration led to effective inhibition of renin activity in HEK-293 cells, along with reduced angiotensin II (Ang II) protein levels in renin-expressing cells. After treatment, the mice’s serum Ang II concentration fell from 16.56 ± 1.70 pg/mL to 10.86 ± 0.68 pg/mL and 9.14 ± 1.31 pg/mL (at two tested doses), and Ang II expression in bone tissue likewise decreased. These changes were accompanied by improvements in trabecular bone density and microstructural integrity at the proximal tibia and distal femur [91]. Moreover, Tanshinone IIA promotes osteogenic differentiation by upregulating Wnt5α and activating the non-canonical Wnt/β-catenin pathway. This non-canonical pathway partially intersects with the canonical Wnt/β-catenin pathway and Tanshinone IIA has some inhibitory effect on excessive β-catenin expression, thereby preventing the pathological progression of tibial dyschondroplasia (TD). This mechanism, supported by increased BMP-2 expression, highlights Tanshinone IIA’s potential value in intervening in TD [92].

In a rheumatoid arthritis (RA) model (adjuvant-induced arthritis in rats), Tanshinone IIA displayed significant anti-inflammatory and anti-osteoclastic effects. In vitro, this compound effectively blocked RANKL-induced osteoclast differentiation. Activity-based protein profiling (ABPP) combined with LC–MS/MS analysis found that Tanshinone IIA covalently binds to the lactate dehydrogenase C subunit (LDHC), inhibiting its enzymatic activity and reducing intracellular ROS accumulation, thereby downregulating osteoclast-specific markers and blocking osteoclast formation and differentiation. This study confirmed that Tanshinone IIA, by interfering with LDHC-mediated ROS generation, has therapeutic potential in preventing RA-associated bone destruction [93].

In OVX mouse models, significant trabecular bone loss is observed by 6 weeks post-surgery, whereas Tanshinone IIA treatment clearly slowed this trabecular bone loss. H&E staining and micro-CT imaging both confirmed this protective effect. Evaluation using multiple bone structural parameters—bone volume/total volume (BV/TV), bone surface area/total volume (BS/TV), trabecular number (Tb.N), trabecular pattern factor (Tb.Pf), and bone mineral density (BMD)—further verified that Tanshinone IIA has a strong inhibitory effect on OVX-induced bone loss [94]. Additionally, in vivo experiments showed that compared to the untreated osteoporosis model group, the Tanshinone IIA–treated group had significant improvements in BMD, bone volume fraction, bone volume, and total volume, indicating that Tanshinone IIA helps promote a dynamic balance in bone remodeling [95]. In a C57BL/6J mouse calvarial osteolysis model induced by polyethylene particles, Tanshinone IIA dose-dependently inhibited bone resorption and osteoclast formation. ELISA results showed that Tanshinone IIA significantly reduced the levels of the bone-specific osteoclast-associated receptor (OSCAR) and C-telopeptide of type I collagen (CTX-1), while elevating OPG levels, thereby reducing peri-implant bone destruction. In summary, Tanshinone IIA shows strong promise in counteracting particle-induced osteolysis and may provide a novel pharmacological strategy for preventing and treating aseptic prosthesis loosening [96].

### 5.2. Effective Concentration Range of Tanshinone IIA in Bone Injury Repair

In in vitro osteogenic models, Tanshinone IIA demonstrates a characteristic biphasic dose–response relationship: low-to-moderate micromolar concentrations stimulate osteogenesis, whereas higher concentrations exert inhibitory effects. In mouse bone marrow–derived mesenchymal stem cells (BMSCs), treatment with 1–5 μM Tanshinone IIA markedly enhances alkaline phosphatase (ALP) activity and matrix mineralization, accompanied by upregulation of osteogenic signaling pathways such as RUNX2, Wnt/β-catenin, and BMP. However, when the concentration is elevated to 20 μM, osteogenic differentiation is suppressed, indicating a ceiling effect detrimental to osteogenesis [97]. Comparable results have been observed in human periodontal ligament stem cells (hPDLSCs), where 5 μM Tanshinone IIA at 21 days produced the most robust increases in ALP activity, mineralized nodule formation, and RUNX2 expression, further supporting a low-to-mid micromolar optimal window. Consistent with these findings, systematic reviews have summarized that the effective in vitro exposure range of Tanshinone IIA in bone-related cells generally spans 10^–8–10^–5 M. Although the precise optimum varies depending on cell type, induction protocol, and culture conditions, a common feature is that exceeding the upper threshold (≥10–20 μM) is frequently associated with reduced differentiation efficiency or overt cytotoxicity [98].

In in vivo bone injury models, an OVX mouse fracture study employed an injectable hydrogel for localized delivery of Tanshinone IIA. During hydrogel preparation, Tanshinone IIA was incorporated at 4 μM, yielding a final working concentration of 2 μM (20 μL) when injected into the medullary cavity. Over a 4-week period, this delivery strategy significantly increased callus bone mineral density (BMD) and bone volume fraction (BV/TV), while improving biomechanical strength. At the early stage of fracture healing, it also reduced TUNEL-positive apoptotic cells and activated the Nrf2/antioxidant defense pathway, indicating that ~2 μM local exposure is sufficient to confer substantial reparative benefits [99].

In summary, current evidence indicates that Tanshinone IIA possesses a narrow yet clearly defined effective concentration window in the context of bone tissue engineering and repair. In vitro, effective concentrations are generally situated within 10^–8–10^–5 M, with 1–5 μM representing a frequently reported pro-osteogenic range, whereas exposure at ≥10–20 μM tends to produce inhibitory or cytotoxic outcomes. In vivo, localized delivery demonstrates that ~2 μM at the fracture site can achieve both structural and biomechanical improvements. Collectively, these findings underscore the importance of dose selection, delivery strategy, and the local microenvironment in determining the biological outcomes of Tanshinone IIA, and highlight the necessity of precisely reporting cell or tissue context, induction conditions, delivery systems, and actual exposure concentrations to ensure reproducibility and cross-study comparability.

### 5.3. Challenges in Drug Delivery: Design and Implementation Strategies

The clinical prospects of Tanshinone IIA are significantly constrained by its poor water solubility, limited biomembrane permeability, and low oral bioavailability. To address these limitations, researchers developed a Tanshinone IIA liposomal formulation aimed at enhancing its in vivo bioavailability and sustained-release profile. The prepared liposomes had a particle size of 150.67 ± 27.23 nm, PDI 0.20 ± 0.015, zeta potential −8.73 ± 2.28 mV, encapsulation efficiency (EE) 70.32 ± 4.04%, and drug loading (DL) 15.63%. qRT-PCR results indicated that in the liposome-treated group, vimentin expression was significantly upregulated while MHCIIB transcript levels were significantly downregulated (*p* < 0.05). Western blot further demonstrated that this formulation enhanced the expression of autophagy-related proteins VPS34, Beclin-1, and LC3B, and reduced the accumulation of p62, suggesting that the liposomes promote muscle injury repair by activating autophagy. Integrating H&E staining, immunohistochemistry, ELISA, and serological analyses, the Tanshinone IIA liposomes effectively promoted desmin synthesis, inhibited type I collagen expression, and significantly decreased levels of inflammatory factors such as TNF-α and IL-6 (*p* < 0.05). Additionally, this formulation lowered malondialdehyde (MDA) content after muscle injury and increased superoxide dismutase (SOD) activity (*p* < 0.05). Therefore, Tanshinone IIA liposomes, through autophagy activation and anti-oxidative stress mechanisms, demonstrated remarkable efficacy in the treatment of acute blunt muscle injury, providing a new therapeutic concept for the sustained delivery of poorly soluble drugs and the repair of tissue injuries [100].

For pulmonary delivery, a Tanshinone IIA–based bioactive nanoemulsion (NE) was designed and evaluated for the first time in an LPS-induced acute lung injury (ALI) rat model. This nanoemulsion was simple to prepare and used excipients with excellent biocompatibility and intrinsic pharmacological activity, achieving outstanding colloidal stability and encapsulation efficiency. Mechanistic studies showed that Tanshinone IIA protects lung tissue by preventing the degradation and shedding of the endothelial glycocalyx, an effect closely related to its antioxidant and anti-inflammatory properties. Incorporating Tanshinone IIA into the nanoemulsion, combined with pharmacologically active excipients (tea tree oil and rhamnolipids), further enhanced its anti-inflammatory and antioxidant effects. Moreover, by virtue of the nano-delivery system’s targeting ability, this approach achieved more effective intervention in ALI [101].

To further address poor oral bioavailability, researchers constructed Tanshinone IIA lipid nanocapsules (LNCs) and optimized their preparation via the phase inversion method, thereby improving their pharmacokinetic performance. The fabricated Tanshinone IIA-LNCs had a particle size of ~70 nm, PDI < 0.2, zeta potential −13.5 mV, encapsulation efficiency ~98%, and drug loading ~2.6 mg/g. In vivo pharmacokinetic studies showed that compared to a traditional suspension, Tanshinone IIA-LNCs increased the oral absorption and exposure (AUC_0–∞) by approximately 3.6-fold (*p* ≤ 0.01), and significantly prolonged the half-life and mean residence time (*p* ≤ 0.01), fully confirming its long-acting release characteristics. This nanocapsule delivery platform provides a reliable technical solution for enhancing the oral bioavailability of Tanshinone IIA [102].

In the context of atherosclerosis therapy, a novel nano-delivery system was developed comprising a metal–(Tanshinone IIA) phenolic network (MPN) combined with reconstituted high-density lipoprotein (rHDL), termed SSPH-MPN@rHDL, to achieve co-delivery of multiple plant-derived active components. This core–shell nanostructure can load both hydrophilic and hydrophobic drugs, remains stable under physiological conditions, and releases its payload responsively in the acidic microenvironment of atherosclerotic plaques. In vitro and in vivo studies demonstrated that this system effectively reduced lipid accumulation within plaques, alleviated oxidative stress, and inhibited inflammatory cytokine expression, thereby slowing the progression of atherosclerosis. This strategy provides a novel avenue for the precision treatment of complex cardiovascular diseases and represents an advance in drug delivery technology [103].

### 5.4. Therapeutic Efficacy and Safety Monitoring of Tanshinone IIA

Tanshinone IIA may effectively suppress chronic inflammatory responses by reducing the levels of inflammatory cytokines. In addition, its free radical–scavenging ability helps mitigate cell damage caused by inflammatory mediators. Observations in inflammatory bowel disease suggest that Tanshinone IIA can ameliorate these pathological features. Studies have shown that patients with ulcerative colitis generally exhibit increased capillary permeability and elevated levels of inflammatory cytokines. Tanshinone IIA, by modulating capillary permeability and reducing the release of inflammatory cytokines, has demonstrated effects consistent with improving these pathological changes. These results suggest that Tanshinone IIA may reduce the secretion of inflammatory factors by inhibiting lymphocyte activity, and importantly, no significant adverse reactions were observed. Despite certain limitations in the current research, the findings indicate that Tanshinone IIA has potential as an adjuvant therapy for ulcerative colitis. In the future, large-scale, multi-center studies will be needed to further verify its efficacy and assess long-term safety [104].

To evaluate the cytotoxicity and genotoxicity of Tanshinone IIA, MTT and hypoxanthine-guanine phosphoribosyltransferase (HPRT) assays have been performed. In vitro cytotoxicity tests showed that the cytotoxic effects observed in Chinese hamster ovary (CHO) cells were consistent with results in human liver cancer HepG2 cells and H9c2 cardiomyocytes. For genotoxicity assessment, changes in HPRT gene mutation frequency in CHO cells after 4 h of Tanshinone IIA exposure were measured. According to relevant guidelines from the Organization for Economic Cooperation and Development (OECD), Tanshinone IIA did not induce gene mutations in this system, indicating low genotoxicity [105].

Regarding lipid metabolism regulation, Tanshinone IIA significantly enhances low-density lipoprotein receptor (LDLR) levels by downregulating proprotein convertase subtilisin/kexin type 9 (PCSK9) expression in human HepG2 hepatocytes, thereby increasing cellular uptake of LDL. By modulating the SREBP2/PCSK9 pathway, Tanshinone IIA not only elevated high-density lipoprotein (HDL) levels in hyperlipidemic rats’ serum, but also reduced hepatic lipid accumulation. Meanwhile, Tanshinone IIA inhibited the expression of sterol regulatory element-binding protein 1 (SREBP1) and its liver X receptor α (LXRα)–mediated transcriptional activity, lowering the expression of lipid-synthesizing enzymes such as fatty acid synthase (FASN), acetyl-CoA carboxylase 1 (ACC1), and stearoyl-CoA desaturase 1 (SCD1). This, in turn, suppressed lipogenesis and reduced lipid accumulation. Additionally, Tanshinone IIA influenced the AMPK/ACC/CPT1 signaling pathway to modulate fatty acid β-oxidation during ischemia, which helps maintain levels of total cholesterol (TC), triglycerides (TG), and free fatty acids (FFA) in the blood. Tanshinone IIA is considered an inhibitor of apolipoprotein B and triglyceride secretion, and it is also a natural antagonist of peroxisome proliferator-activated receptor γ (PPARγ) [106].

In cardiovascular studies, Tanshinone IIA has been confirmed to induce coronary artery dilation in mice, rats, and pigs, involving factors such as the vascular endothelium, nitric oxide (NO), epoxyeicosatrienoic acids (EETs), and large-conductance Ca^2+-activated K^+ channels (BK_Ca). Moreover, Tanshinone IIA significantly reduced atherosclerotic plaque size and lesion area in *ApoE*^−/−^ mice by decreasing superoxide anion production, inhibiting LDL oxidation, lowering cholesterol levels, and reducing pro-inflammatory cytokines. Further research showed that Tanshinone IIA causes relaxation of the thoracic aorta in male Sprague–Dawley rats, an effect dependent on NO and estrogen signaling pathways. Although some studies have examined the effects of whole Danshen root preparations on coronary and femoral arteries, the effects of Tanshinone IIA and its water-soluble derivatives on peripheral vascular resistance have not been fully explored. Our group’s research indicates that Tanshinone IIA induces dilation of the mesenteric arteries in male Sprague–Dawley rats, an effect mediated mainly by small- and intermediate-conductance Ca^2+^-activated K^⁺^ channels (SK_Ca and IK_Ca) and independent of the NO pathway [107].

In the treatment of myocardial ischemia and hypoxia, Tanshinone IIA has shown significant efficacy. It can effectively inhibit angiotensin II production and reduce cardiac remodeling, markedly improving clinical symptoms of angina pectoris. By intravenous infusion, Tanshinone IIA can maintain stable plasma concentrations, improve blood circulation, dilate coronary arteries, and lower heart rate, thereby improving angina-related indices and hemorheological parameters, optimizing blood lipid profiles, and reducing C-reactive protein (CRP) levels. A related systematic review pooled all Chinese and English studies of Tanshinone IIA combined with conventional therapy for unstable angina pectoris (UAP). After strict screening and meta-analysis, it reached more reliable conclusions based on large sample sizes, which are more persuasive and credible than individual studies [108].

Although Tanshinone IIA has shown significant efficacy in the treatment of UAP, its adverse reactions merit attention. Fifteen cases of adverse reactions have been reported in the literature, mainly manifesting as facial flushing, dizziness, headache, fever, and rash. These adverse reactions may be related to sensitizing substances generated during Danshen extraction or other components in the formulation process. Tanshinone IIA injection solutions may undergo sulfonation upon long-term storage, which can trigger allergic responses and adverse events. Therefore, during clinical application, healthcare professionals should closely monitor for adverse reactions and properly dilute the drug according to the instructions, while avoiding inappropriate combinations with other medications [109].

## 6. Conclusions and Future Perspectives

Bone injuries are common clinical problems that are often difficult to treat. Especially under pathological conditions such as delayed fracture healing, non-union, osteoporotic fractures, and impaired bone regeneration (e.g., after osteomyelitis), there is a higher demand for effective bone tissue repair strategies. In recent years, natural products have received extensive attention in the field of bone repair and regeneration, among which Tanshinone IIA is considered a highly promising candidate due to its wide availability, well-defined structure, and notable bioactivity. A wealth of experimental data indicates that Tanshinone IIA not only possesses multiple effects—anti-inflammatory, antioxidant, immunomodulatory, anti-apoptotic, and anti-osteoclastic—but can also drive the differentiation of BMSCs into the osteoblast lineage through numerous signaling pathways (e.g., Wnt/β-catenin, PI3K/Akt, MAPK, NF-κB, BMP/Smad), while inhibiting their differentiation into adipocytes, thereby significantly promoting bone formation and slowing bone loss. Additionally, Tanshinone IIA can regulate osteoclast formation and function, balancing the dynamic interplay between bone resorption and bone formation during bone remodeling. More importantly, Tanshinone IIA has shown promising therapeutic prospects for inflammatory bone diseases (such as RA-induced bone erosion or diabetic bone injury) and hormone-related bone disorders (such as glucocorticoid-induced osteoporosis).

Despite the encouraging findings so far, research on Tanshinone IIA in bone tissue repair still faces many challenges and gaps. Future studies should be advanced and deepened in the following aspects:

Clarify key targets and signaling networks: Most current studies focus on Tanshinone IIA’s effects on single pathways or single cell types, and a systematic analysis is lacking. By employing multi-omics approaches (transcriptomics, proteomics, metabolomics, etc.) combined with high-throughput screening and gene editing tools (e.g., CRISPR/Cas9), researchers should identify the core targets and regulatory networks of Tanshinone IIA, providing a solid molecular basis for understanding its pharmacological mechanisms.

Improve pharmacokinetics and targeting: Tanshinone IIA’s pharmacokinetic profile and tissue distribution are not yet fully understood, and its poor solubility and low oral bioavailability are major bottlenecks limiting clinical translation. Advanced nano-delivery technologies (such as liposomes, nanoemulsions, solid lipid nanoparticles, etc.) should be utilized to improve its stability and targeting. In particular, developing bone-targeted delivery systems with controlled release could increase the effective concentration of Tanshinone IIA locally in bone tissue and achieve precision therapy.

Advance preclinical research in relevant models: Building on existing cell and small-animal experiments, the translation of Tanshinone IIA into large animal models and comprehensive preclinical studies should be accelerated. It is especially important to evaluate its efficacy in different types of bone injury models (such as load-bearing fractures, large segmental bone defects, bone regeneration impairments post-osteomyelitis, etc.) to provide evidence that closely reflects clinical scenarios. Notably, Tanshinone IIA’s capacity to promote bone repair in the context of chronic diseases or metabolic disorders remains unclear, and its effectiveness and safety in complex pathological environments require further evaluation.

Explore combination therapies and biomaterials: Research on combining Tanshinone IIA with other therapies or biomaterials should be strengthened. For instance, co-delivery of Tanshinone IIA with growth factors (e.g., BMP-2, VEGF), stem cells, or biomimetic scaffold materials to construct composite repair systems could integrate biochemical cues with structural support. Such combined approaches may overcome the limitations of single-drug therapy and enhance the clinical applicability of bone tissue engineering strategies.

Standardize formulation and safety evaluations: As an active ingredient derived from traditional medicine, the quality control standards, formulation development, dose optimization, and safety evaluation of Tanshinone IIA need to be standardized. Systematic toxicological studies should be conducted and a comprehensive preclinical safety assessment system should be established to avoid potential toxic side effects. Additionally, monitoring the impact of long-term use is crucial, with particular attention to inter-individual variability in patients with chronic diseases or in the elderly, to ensure the sustainable and safe clinical application of Tanshinone IIA.

In conclusion, Tanshinone IIA has demonstrated multi-target, multi-pathway efficacy with low toxicity and high therapeutic potential in the field of bone injury repair, positioning it as a highly promising natural therapeutic agent. However, progressing from laboratory research to clinical application will require multidisciplinary collaboration involving fields such as medicinal chemistry, bone biology, materials science, pharmaceutics, and translational medicine. With ongoing advances in precision medicine and bioengineering, Tanshinone IIA is expected to develop into a novel, effective, and safe natural intervention for orthopedic diseases, potentially bringing transformative progress to clinical bone repair.

## Figures and Tables

**Table 1:** Tanshinone IIA acts on cellular targets—mechanisms.

Cell/Disease-Related Model	Major Effect	Mechanism/Pathway Axis
BMSCs (under hypoxia/osteogenic induction)	↑ ALP and mineralization; ↓ adipogenic differentiation	Synergistic activation of Akt/CREB and TGF-β/Smad3 signaling
Osteoblasts (e.g., MC3T3-E1)	Antioxidative/anti-apoptotic; maintenance of osteogenic phenotype	Inhibits Nox4-ROS and NF-κB; activates Nrf2 antioxidant pathway
Osteoclast precursors/osteoclasts (RANKL-induced)	Inhibits differentiation and bone resorption	↓ RANKL/↑ OPG; suppression of c-Fos/NFATc1 and NF-κB
Chondrocytes (CHON-001; human chondrocytes)	Anti–IL-1β inflammation and protection from matrix degradation	↓ FBXO11 → inhibition of PI3K/Akt and NF-κB; ↑ NEAT1_2 to maintain chondrocyte phenotype
Synovial fibroblast-like cells (RA-FLS)	Anti-TNF-α inflammatory cascade	↑ miR-124-3p; ↓ miR-132-3p/miR-155-5p
Endothelial cells (HUVEC; CD31^hi Emcn^hi)	Context-dependent regulation of angiogenesis	↑ HIF-1α/VEGF–VEGFR2 signaling or inhibition of OA-related VEGFR2–MAPK axis
Macrophages/immune cells	Anti-inflammatory and immunometabolic reprogramming	Inhibits SDH–HIF-1α and NLRP3; ↑ NAD⁺/Sirt2; activates Nrf2/HO-1
Osteosarcoma/bone-related tumor cells	Induces apoptosis; inhibits proliferation	Mitochondrial dysfunction; JNK-dependent and extrinsic apoptosis pathways

Arrows: ↑ increase/upregulation/activation; ↓ decrease/downregulation/inhibition; → leads to/results in.

**Table 2:** Danshenone II A Modulation of Pathways and Targets in Bone Disease Models.

Animal Model	Key Target(s)	Mechanism/Pathway
Osteoarthritis (rat; ACLT + MMx)	MMPs ↓; TIMPs ↑; BMP ↑; TGF-β ↑; IL-1β/TNF-α/NO ↓	Inhibits MMPs; enhances TIMPs and anabolic factors; suppresses inflammatory mediators
Osteoporosis (rat; OVX—alveolar bone/BMSCs)	PHGDH ↑ (via promoter demethylation)	Restores PHGDH expression; delays BMSC senescence; preserves stemness
Diabetic bone loss (mouse; STZ)	Renin activity ↓; Ang II ↓	Inhibits renin activity and Ang II signaling in bone
Tibial dyschondroplasia (TD) (experimental model)	Wnt5α ↑; BMP-2 ↑; moderated β-catenin	Activates noncanonical Wnt/β-catenin; restrains excessive canonical β-catenin
Rheumatoid arthritis–related bone loss (rat; adjuvant-induced)	LDHC inhibition; osteoclast markers ↓	Binds/inhibits LDHC → reduces ROS → blocks RANKL-induced osteoclastogenesis
Osteoporosis (mouse; OVX—general bone loss)	Bone indices improved: BV/TV, BS/TV, Tb.N, Tb.Pf, BMD	Exerts antiresorptive and osteoprotective effects on trabecular bone
Particle-induced calvarial osteolysis (mouse; polyethylene particles)	OPG ↑; OSCAR ↓; CTX-1 ↓	Inhibits osteoclastogenesis and bone resorption around implant
Fracture repair (mouse; OVX, hydrogel delivery)	Nrf2 ↑; apoptosis ↓	Activates Nrf2 antioxidant pathway; reduces TUNEL-positive cells

Arrows: ↑ increase/upregulation/activation; ↓ decrease/downregulation/inhibition; → leads to/results in.

## Data Availability

Not applicable.

## References

[1] Muñoz M.B., Robinson K., Shibli-Rahhal A.M. (2020). Bone Health and Osteoporosis Prevention and Treatment. Clin. Obs. Gynecol..

[2] LeBoff M.S., Greenspan S.L., Insogna K.L., Lewiecki E.M., Saag K.G., Singer A.J., Siris E.S. (2022). The clinician’s guide to prevention and treatment of osteoporosis. Osteoporos. Int..

[3] Brown J.P. (2021). Long-Term Treatment of Postmenopausal Osteoporosis. Endocrinol. Metab..

[4] Arceo-Mendoza R.M., Camacho P.M. (2021). Postmenopausal Osteoporosis: Latest Guidelines. Endocrinol. Metab. Clin. N. Am..

[5] Ye Z., Liu Y., Song J., Gao Y., Fang H., Hu Z., Zhang M., Liao W., Cui L., Liu Y. (2023). Expanding the therapeutic potential of Salvia miltiorrhiza: A review of its pharmacological applications in musculoskeletal diseases. Front. Pharmacol..

[6] Li Y., Wang W., Xu W. (2025). Mechanisms and new advances in the efficacy of plant active ingredients in tendon-bone healing. J. Orthop. Surg. Res..

[7] Guo R., Li L., Su J., Li S., Duncan S.E., Liu Z., Fan G. (2020). Pharmacological Activity and Mechanism of Tanshinone IIA in Related Diseases. Drug Des. Dev. Ther..

[8] Chen Z., Feng H., Peng C., Zhang Z., Yuan Q., Gao H., Tang S., Xie C. (2023). Renoprotective Effects of Tanshinone IIA: A Literature Review. Molecules.

[9] Liu Q., Li X., Luo Y. (2024). Tanshinone IIA delays liver aging by modulating oxidative stress. Front. Pharmacol..

[10] Ekeuku S.O., Pang K.L., Chin K.Y. (2021). The Skeletal Effects of Tanshinones: A Review. Molecules.

[11] Zeng H., Su S., Xiang X., Sha X., Zhu Z., Wang Y., Guo S., Yan H., Qian D., Duan J. (2017). Comparative Analysis of the Major Chemical Constituents in Salvia miltiorrhiza Roots, Stems, Leaves and Flowers during Different Growth Periods by UPLC-TQ-MS/MS and HPLC-ELSD Methods. Molecules.

[12] Zhong C., Lin Z., Ke L., Shi P., Li S., Huang L., Lin X., Yao H. (2021). Recent Research Progress (2015–2021) and Perspectives on the Pharmacological Effects and Mechanisms of Tanshinone IIA. Front. Pharmacol..

[13] Zhou Z.Y., Zhao W.R., Zhang J., Chen X.L., Tang J.Y. (2019). Sodium tanshinone IIA sulfonate: A review of pharmacological activity and pharmacokinetics. Biomed. Pharmacother..

[14] Xu J., Zhang C., Shi X., Li J., Liu M., Jiang W., Fang Z. (2019). Efficacy and Safety of Sodium Tanshinone IIA Sulfonate Injection on Hypertensive Nephropathy: A Systematic Review and Meta-Analysis. Front. Pharmacol..

[15] Zheng Z., Ke L., Ye S., Shi P., Yao H. (2024). Pharmacological Mechanisms of Cryptotanshinone: Recent Advances in Cardiovascular, Cancer, and Neurological Disease Applications. Drug Des. Dev. Ther..

[16] Bi Z., Wang Y., Zhang W. (2021). A comprehensive review of tanshinone IIA and its derivatives in fibrosis treatment. Biomed. Pharmacother..

[17] Xiao Z., Liu W., Mu Y.P., Zhang H., Wang X.N., Zhao C.Q., Chen J.M., Liu P. (2020). Pharmacological Effects of Salvianolic Acid B Against Oxidative Damage. Front. Pharmacol..

[18] He G., Chen G., Liu W., Ye D., Liu X., Liang X., Song J. (2023). Salvianolic Acid B: A Review of Pharmacological Effects, Safety, Combination Therapy, New Dosage Forms, and Novel Drug Delivery Routes. Pharmaceutics.

[19] Xing L., Tan Z.R., Cheng J.L., Huang W.H., Zhang W., Deng W., Yuan C.S., Zhou H.H. (2017). Bioavailability and pharmacokinetic comparison of tanshinones between two formulations of Salvia miltiorrhiza in healthy volunteers. Sci. Rep..

[20] Yang Y., Yang J., Fu W., Zhou P., He Y., Fang M., Wan H., Zhou H. (2022). Pharmacokinetic Comparison of Nine Bioactive Compounds of Guanxinshutong Capsule in Normal and Acute Myocardial Infarction Rats. Eur. J. Drug. Metab. Pharmacokinet..

[21] Bhadoriya A., Shah P.A., Shrivastav P.S., Bharwad K.D., Singhal P. (2019). Determination of terbinafine in human plasma using UPLC-MS/MS: Application to a bioequivalence study in healthy subjects. Biomed. Chromatogr..

[22] Wang D., Yu W., Cao L., Xu C., Tan G., Zhao Z., Huang M., Jin J. (2020). Comparative pharmacokinetics and tissue distribution of cryptotanshinone, tanshinone IIA, dihydrotanshinone I, and tanshinone I after oral administration of pure tanshinones and liposoluble extract of Salvia miltiorrhiza to rats. Biopharm. Drug Dispos..

[23] Ashour A.A., Ramadan A.A., Abdelmonsif D.A., El-Kamel A.H. (2020). Enhanced oral bioavailability of Tanshinone IIA using lipid nanocapsules: Formulation, in-vitro appraisal and pharmacokinetics. Int. J. Pharm..

[24] Yan H.-M., Sun E., Cui L., Jia X.-B., Jin X. (2015). Improvement in oral bioavailability and dissolution of tanshinone IIA by preparation of solid dispersions with porous silica. J. Pharm. Pharmacol..

[25] Li Z., Zhang Y., Zhang K., Wu Z., Feng N. (2018). Biotinylated-lipid bilayer coated mesoporous silica nanoparticles for improving the bioavailability and anti-leukaemia activity of Tanshinone IIA. Artif. Cells Nanomed. Biotechnol..

[26] Song L., Zhang W., Tang S.Y., Luo S.M., Xiong P.Y., Liu J.Y., Hu H.C., Chen Y.Q., Jia B., Yan Q.H. (2024). Natural products in traditional Chinese medicine: Molecular mechanisms and therapeutic targets of renal fibrosis and state-of-the-art drug delivery systems. Biomed. Pharmacother..

[27] Wang J., Kong L., Guo R.B., He S.Y., Liu X.Z., Zhang L., Liu Y., Yu Y., Li X.T., Cheng L. (2022). Multifunctional icariin and tanshinone IIA co-delivery liposomes with potential application for Alzheimer’s disease. Drug Deliv..

[28] A Ashour A., El-Kamel A.H., A Abdelmonsif D., Khalifa H.M., A Ramadan A. (2021). Modified Lipid Nanocapsules for Targeted Tanshinone IIA Delivery in Liver Fibrosis. Int. J. Nanomed..

[29] Cai C., Liu K., Yang D., Wu J., Peng Z., Wang Y., Xi J., Xie F., Li X. (2025). The nanocrystal-loaded liposome of tanshinone IIA with high drug loading and stability towards efficient liver fibrosis reversion. Nanomedicine.

[30] Zhang Y., Jiang P., Ye M., Kim S.-H., Jiang C., Lü J. (2012). Tanshinones: Sources, pharmacokinetics and anti-cancer activities. Int. J. Mol. Sci..

[31] Huang C.Y., Chiu T.L., Kuo S.J., Chien S.Y., Chen D.R., Su C.C. (2013). Tanshinone IIA inhibits the growth of pancreatic cancer BxPC-3 cells by decreasing protein expression of TCTP, MCL-1 and Bcl-xL. Mol. Med. Rep..

[32] Ouyang Ouyang D.-S., Huang W.H., Chen D., Zhang W., Tan Z.R., Peng J.B., Wang Y.C., Guo Y., Hu D.L., Xiao J. (2016). Kinetics of cytochrome P450 enzymes for metabolism of sodium tanshinone IIA sulfonate in vitro. Chin. Med..

[33] Liang S., Wang Z., Yuan J., Zhang J., Dai X., Qin F., Zhang J., Sun Y. (2019). Rapid Identification of Tanshinone IIA Metabolites in an Amyloid-β(1–42) Induced Alzherimer’s Disease Rat Model using UHPLC-Q-Exactive Qrbitrap Mass Spectrometry. Molecules.

[34] Wang Y., Yan J., Li S., Cai X., Wang W., Luo K., Huang D., Gao J. (2014). Pharmacokinetics and tissue distribution study of tanshinone IIA after oral administration of Bushen Huoxue Qubi granules to rats with blood stasis syndrome. Pharmacogn. Mag..

[35] Sudha S., Upmanyu A., Saraswat D., Singh M. (2025). Pharmacological impacts of tanshinone on osteogenesis and osteoclastogenesis: A review. Naunyn Schmiedeberg’s Arch. Pharmacol..

[36] Takayanagi H., Kim S., Koga T., Nishina H., Isshiki M., Yoshida H., Saiura A., Isobe M., Yokochi T., Inoue J.I. (2002). Induction and activation of the transcription factor NFATc1 (NFAT2) integrate RANKL signaling in terminal differentiation of osteoclasts. Dev. Cell.

[37] de Molon R.S. (2025). Therapeutic Potential of Tanshinones in Osteolytic Diseases: From Molecular and Cellular Pathways to Preclinical Models. Dent. J..

[38] Li Y., Zhang L., Wang J., Zheng Y., Cui J., Yuan G. (2021). Tanshinone IIA attenuates polyethylene-induced osteolysis in a mouse model: The key role of miR-155-5p/FOXO3 axis. J. Funct. Foods.

[39] Li J., He C., Tong W., Zou Y., Li D., Zhang C., Xu W. (2015). Tanshinone IIA blocks dexamethasone-induced apoptosis in osteoblasts through inhibiting Nox4-derived ROS production. Int. J. Clin. Exp. Pathol..

[40] Wang W., Wu H., Feng S., Huang X., Xu H., Shen X., Fu Y., Fang S. (2024). Tanshinone IIA promotes osteogenic differentiation potential and suppresses adipogenic differentiation potential of bone marrow mesenchymal stem cells. Mol. Med. Rep..

[41] Cheng S., Hu X., Sun K., Huang Z., Zhao Y., Sun Y., Zeng B., Wang J., Zhao D., Lu S. (2024). Local Application of Tanshinone IIA protects mesenchymal stem cells from apoptosis and promotes fracture healing in ovariectomized mice. J. Orthop. Surg. Res..

[42] Li X., Yang X., Liu Z., Liu H., Lv H., Li X., Xu X., Shen Y. (2025). Tanshinone I IA Reverses Osteogenic Differentiation of Bone Marrow Mesenchymal Stromal Cells Impaired by Glucocorticoids via the ERK1/2-CREB Signaling Pathway. Chem. Biol. Drug Des..

[43] Xu J., Zhi X., Zhang Y., Ding R. (2024). Tanshinone IIA alleviates IL-1β-induced chondrocyte apoptosis and inflammation by regulating FBXO11 expression. Clinics.

[44] Sun J., Chen W., Zhou Z., Chen X., Zuo Y., He J., Liu H. (2023). Tanshinone IIA Facilitates Efficient Cartilage Regeneration under Inflammatory Factors Caused Stress via Upregulating LncRNA NEAT1_2. Biomedicines.

[45] Li H.Z., Han D., Ao R.F., Cai Z.H., Zhu G.Z., Wu D.Z., Gao J.W., Zhuang J.S., Tu C., Zhao K. (2024). Tanshinone IIA attenuates osteoarthritis via inhibiting aberrant angiogenesis in subchondral bone. Arch. Biochem. Biophys..

[46] Zhou B., Li L.H., Tan L.M., Luo W.B., Xiong H., Lu X.L., Liu D., Li W.Y., Guo Y.X., Tang Z. (2021). Tanshinone IIA Ameliorates Inflammation Response in Osteoarthritis via Inhibition of miR-155/FOXO3 Axis. Pharmacology.

[47] Feng M., Peng H., Yao R., Zhang Z., Mao G., Yu H., Qiu Y. (2020). Inhibition of cellular communication network factor 1 (CCN1)-driven senescence slows down cartilage inflammaging and osteoarthritis. Bone.

[48] Ding B., Lin C., Liu Q., He Y., Ruganzu J.B., Jin H., Peng X., Ji S., Ma Y., Yang W. (2020). Tanshinone IIA attenuates neuroinflammation via inhibiting RAGE/NF-κB signaling pathway in vivo and in vitro. J. Neuroinflammation.

[49] Fu K., Feng C., Shao L., Mei L., Cao R. (2021). Tanshinone IIA exhibits anti-inflammatory and antioxidative effects in LPS-stimulated bovine endometrial epithelial cells by activating the Nrf2 signaling pathway. Res. Vet. Sci..

[50] Yang L., Zhou G., Liu J., Song J., Zhang Z., Huang Q., Wei F. (2021). Tanshinone I and Tanshinone IIA/B attenuate LPS-induced mastitis via regulating the NF-κB. Biomed. Pharmacother..

[51] Carpi S., Quarta S., Doccini S., Saviano A., Marigliano N., Polini B., Massaro M., Carluccio M.A., Calabriso N., Wabitsch M. (2023). Tanshinone IIA and Cryptotanshinone Counteract Inflammation by Regulating Gene and miRNA Expression in Human SGBS Adipocytes. Biomolecules.

[52] Liu Q.Y., Zhuang Y., Song X.R., Niu Q., Sun Q.S., Li X.N., Li N., Liu B.L., Huang F., Qiu Z.X. (2021). Tanshinone IIA prevents LPS-induced inflammatory responses in mice via inactivation of succinate dehydrogenase in macrophages. Acta Pharmacol. Sin..

[53] Zhang B., Yu P., Su E., Jia J., Zhang C., Xie S., Huang Z., Dong Y., Ding J., Zou Y. (2022). Sodium Tanshinone IIA Sulfonate Improves Adverse Ventricular Remodeling Post-MI by Reducing Myocardial Necrosis, Modulating Inflammation, and Promoting Angiogenesis. Curr. Pharm. Des..

[54] Li F., Zhao S., Fang Q., Qiao Z., Meng Y., Jin Q., Zong L., Shui L., Chen S., Han H. (2025). Tanshinone IIA improved psychological stress-induced embryo implantation disorders by inhibiting GC/GR signaling and promoting angiogenesis. Phytomedicine.

[55] Wang X., Wu C. (2022). Tanshinone IIA improves cardiac function via regulating miR-499–5p dependent angiogenesis in myocardial ischemic mice. Microvasc. Res..

[56] Xu J., Zhang P., Chen Y., Xu Y., Luan P., Zhu Y., Zhang J. (2021). Sodium tanshinone IIA sulfonate ameliorates cerebral ischemic injury through regulation of angiogenesis. Exp. Ther. Med..

[57] Lou G., Hu W., Wu Z., Xu H., Yao H., Wang Y., Huang Q., Wang B., Wen L., Gong D. (2020). Tanshinone II A attenuates vascular remodeling through klf4 mediated smooth muscle cell phenotypic switching. Sci. Rep..

[58] Li H., Hu P., Zou Y., Yuan L., Xu Y., Zhang X., Luo X., Zhang Z. (2023). Tanshinone IIA and hepatocellular carcinoma: A potential therapeutic drug. Front. Oncol..

[59] Li Q., Huang D., Liao W., Su X., Li J., Zhang J., Fang M., Liu Y. (2024). Tanshinone IIA regulates CCl_4_ induced liver fibrosis in C57BL/6J mice via the PI3K/Akt and Nrf2/HO-1 signaling pathways. J. Biochem. Mol. Toxicol..

[60] Zhang Y., Wang J., Yang S., Kou H., Liu P. (2025). Tanshinone IIA alleviate atherosclerosis and hepatic steatosis via down-regulation of MAPKs/NF-κB signaling pathway. Int. Immunopharmacol..

[61] Wang D., Tan Q., Zheng Q., Ma Y., Feng L. (2025). Tanshinone IIA attenuates hepatic stellate cell activation, oxidative stress, and liver fibrosis by inhibiting YAP signaling. Eur. J. Histochem..

[62] Qin C., Liu S., Zhou S., Xia X., Hu J., Yu Y., Ma D. (2023). Tanshinone IIA promotes vascular normalization and boosts Sorafenib’s anti-hepatoma activity via modulating the PI3K-AKT pathway. Front. Pharmacol..

[63] Tian W., Song P., Zang J., Zhao J., Liu Y., Wang C., Fang H., Wang H., Zhao Y., Liu X. (2025). Tanshinone IIA, a component of Salvia miltiorrhiza Bunge, attenuated sepsis-induced liver injury via the SIRT1/Sestrin2/HO-1 signaling pathway. J. Ethnopharmacol..

[64] Wu Q., Guan Y.B., Zhang K.J., Li L., Zhou Y. (2023). Tanshinone IIA mediates protection from diabetes kidney disease by inhibiting oxidative stress induced pyroptosis. J. Ethnopharmacol..

[65] Li Y., Wu T., Li H., Liu M., Xu H. (2024). Tanshinone IIA Promoted Autophagy and Inhibited Inflammation to Alleviate Podocyte Injury in Diabetic Nephropathy. Diabetes Metab. Syndr. Obes..

[66] Fan Y., Kang S., Shao T., Xu L., Chen J. (2024). Activation of SIRT3 by Tanshinone IIA ameliorates renal fibrosis by suppressing the TGF-β/TSP-1 pathway and attenuating oxidative stress. Cell. Signal..

[67] Zhang L., Yang F. (2022). Tanshinone IIA improves diabetes-induced renal fibrosis by regulating the miR-34-5p/Notch1 axis. Food Sci. Nutr..

[68] Zhang X., Long F., Li R., Yang Y., Wang T., He Q., Xu M., Wang L., Jiang X. (2022). Tanshinone IIA prevents acetaminophen-induced nephrotoxicity through the activation of the Nrf2-Mrp2/4 pathway in mice. Env. Toxicol..

[69] Xu L., Xu Y., Zhu Z., Gu H., Chen C., Chen J. (2021). Tanshinone IIA attenuates renal injury during hypothermic preservation via the MEK/ERK1/2/GSK-3β pathway. BMC Complement. Med. Ther..

[70] Hu T., Zou H.-X., Le S.Y., Wang Y.R., Qiao Y.M., Yuan Y., Liu J.C., Lai S.Q., Huang H. (2023). Tanshinone IIA confers protection against myocardial ischemia/reperfusion injury by inhibiting ferroptosis and apoptosis via VDAC1. Int. J. Mol. Med..

[71] Chai R., Ye Z., Xue W., Shi S., Wei Y., Hu Y., Wu H. (2023). Tanshinone IIA inhibits cardiomyocyte pyroptosis through TLR4/NF-κB p65 pathway after acute myocardial infarction. Front. Cell Dev. Biol..

[72] Yan N., Xiao C., Wang X., Xu Z., Yang J. (2022). Tanshinone IIA from Salvia miltiorrhiza exerts anti-fibrotic effects on cardiac fibroblasts and rat heart tissues by suppressing the levels of pro-fibrotic factors: The key role of miR-618. J. Food Biochem..

[73] Chen R., Chen W., Huang X., Rui Q. (2021). Tanshinone IIA attenuates heart failure via inhibiting oxidative stress in myocardial infarction rats. Mol. Med. Rep..

[74] Li D., Yang Z., Gao S., Zhang H., Fan G. (2022). Tanshinone IIA ameliorates myocardial ischemia/reperfusion injury in rats by regulation of NLRP3 inflammasome activation and Th17 cells differentiation. Acta Cir. Bras..

[75] Wang X., Wang W.M., Han H., Zhang Y., Liu J.L., Yu J.Y., Liu H.M., Liu X.T., Shan H., Wu S.C. (2022). Tanshinone IIA protected against lipopolysaccharide-induced brain injury through the protective effect of the blood-brain barrier and the suppression of oxidant stress and inflammatory response. Food Funct..

[76] Su W., Lv M., Wang D., He Y., Han H., Zhang Y., Zhang X., Lv S., Yao L., Zhang F. (2024). Tanshinone IIA Alleviates Traumatic Brain Injury by Reducing Ischemia-Reperfusion via the miR-124-5p/FoxO1 Axis. Mediat. Inflamm..

[77] Song Z., Feng J., Zhang Q., Deng S., Yu D., Zhang Y., Li T. (2021). Tanshinone IIA Protects Against Cerebral Ischemia Reperfusion Injury by Regulating Microglial Activation and Polarization via NF-κB Pathway. Front. Pharmacol..

[78] Ma H., Hu Z.C., Long Y., Cheng L.C., Zhao C.Y., Shao M.K. (2022). Tanshinone IIA Microemulsion Protects against Cerebral Ischemia Reperfusion Injury via Regulating H3K18ac and H4K8ac In Vivo and In Vitro. Am. J. Chin. Med..

[79] Xie X., Xu Y., Zhou X., Su P., Jiang X., Jin Z. (2023). The protective effect of an extract of Salvia miltiorrhiza Bunge (Danshen) on cerebral ischemic injury in animal models: A systematic review and meta-analysis. J. Ethnopharmacol..

[80] Sherawat K., Mehan S. (2023). Tanshinone-IIA mediated neuroprotection by modulating neuronal pathways. Naunyn Schmiedeberg’s Arch. Pharmacol..

[81] Liu X.Q., Hu T., Wu G.L., Qiao L.J., Cai Y.F., Wang Q., Zhang S.J. (2024). Tanshinone IIA, the key compound in Salvia miltiorrhiza, improves cognitive impairment by upregulating Aβ-degrading enzymes in APP/PS1 mice. Int. J. Biol. Macromol..

[82] Jin H., Peng X., He Y., Ruganzu J.B., Yang W. (2020). Tanshinone IIA suppresses lipopolysaccharide-induced neuroinflammatory responses through NF-κB/MAPKs signaling pathways in human U87 astrocytoma cells. Brain Res. Bull..

[83] Jiang P., Li C., Xiang Z., Jiao B. (2014). Tanshinone IIA reduces the risk of Alzheimer’s disease by inhibiting iNOS, MMP-2 and NF-κBp65 transcription and translation in the temporal lobes of rat models of Alzheimer’s disease. Mol. Med. Rep..

[84] Geng L., Liu W., Chen Y. (2019). Tanshinone IIA attenuates Aβ-induced neurotoxicity by down-regulating COX-2 expression and PGE2 synthesis via inactivation of NF-κB pathway in SH-SY5Y cells. J. Biol. Res..

[85] Lin C.Y., Chang T.W., Hsieh W.H., Hung M.C., Lin I.H., Lai S.C., Tzeng Y.J. (2016). Simultaneous induction of apoptosis and necroptosis by Tanshinone IIA in human hepatocellular carcinoma HepG2 cells. Cell Death Discov..

[86] Zhang J., Wang J., Jiang J.Y., Liu S.D., Fu K., Liu H.Y. (2014). Tanshinone IIA induces cytochrome c-mediated caspase cascade apoptosis in A549 human lung cancer cells via the JNK pathway. Int. J. Oncol..

[87] Huang S.T., Huang C.C., Huang W.L., Lin T.K., Liao P.L., Wang P.W., Liou C.W., Chuang J.H. (2017). Tanshinone IIA induces intrinsic apoptosis in osteosarcoma cells both in vivo and in vitro associated with mitochondrial dysfunction. Sci. Rep..

[88] Fang Z., Zhang M., Liu J.N., Zhao X., Zhang Y.Q., Fang L. (2020). Tanshinone IIA: A Review of its Anticancer Effects. Front. Pharmacol..

[89] Jia P.T., Zhang X.L., Zuo H.N., Lu X., Li L. (2017). Articular cartilage degradation is prevented by tanshinone IIA through inhibiting apoptosis and the expression of inflammatory cytokines. Mol. Med. Rep..

[90] Wang L., Cheng L., Zhang B., Wang N., Wang F. (2019). Tanshinone prevents alveolar bone loss in ovariectomized osteoporosis rats by up-regulating phosphoglycerate dehydrogenase. Toxicol. Appl. Pharmacol..

[91] Zhang J., Cai Z., Yang M., Tong L., Zhang Y. (2020). Inhibition of tanshinone IIA on renin activity protected against osteoporosis in diabetic mice. Pharm. Biol..

[92] Yang H., Zhang H., Tong X., Zhang J., Shen Y. (2019). Recovery of chicken growth plate by TanshinoneⅡA through wnt/β-catenin pathway in thiram-induced Tibial Dyschondroplasia. Ecotoxicol. Env. Saf..

[93] Peng Q., Wang J., Han M., Zhao M., Li K., Lu T., Guo Q., Jiang Q. (2023). Tanshinone IIA inhibits osteoclastogenesis in rheumatoid arthritis via LDHC-regulated ROS generation. Chin. Med..

[94] Cheng L., Zhou S., Zhao Y., Sun Y., Xu Z., Yuan B., Chen X. (2018). Tanshinone IIA attenuates osteoclastogenesis in ovariectomized mice by inactivating NF-kB and Akt signaling pathways. Am. J. Transl. Res..

[95] Wang Y., Liu L., Qu Z., Wang D., Huang W., Kong L., Yan L. (2022). Tanshinone Ameliorates Glucocorticoid-Induced Bone Loss via Activation of AKT1 Signaling Pathway. Front. Cell Dev. Biol..

[96] Yao J., Ma S., Feng W., Wei Y., Lu H., Zhong G., Wu Z., Wang H., Su W., Li J. (2018). Tanshinone IIA protects against polyethylene particle-induced osteolysis response in a mouse calvarial model. Int. J. Clin. Exp. Pathol..

[97] Qian K., Xu H., Dai T., Shi K. (2015). Effects of Tanshinone IIA on osteogenic differentiation of mouse bone marrow mesenchymal stem cells. Naunyn Schmiedeberg’s Arch. Pharmacol..

[98] Liu X., Niu Y., Xie W., Wei D., Du Q. (2019). Tanshinone IIA promotes osteogenic differentiation of human periodontal ligament stem cells via ERK1/2-dependent Runx2 induction. Am. J. Transl. Res..

[99] Kwak H.B., Yang D., Ha H., Lee J.H., Kim H.N., Woo E.R., Lee S., Kim H.H., Lee Z.H. (2006). Tanshinone IIA inhibits osteoclast differentiation through down-regulation of c-Fos and NFATc1. Exp. Mol. Med..

[100] Li Z., Li Z., Wang B., Liu J. (2022). Influence of release rate, dose and co-administration on pharmacokinetics, pharmacodynamics and PK-PD relationship of tanshinone IIA and tanshinol. Eur. J. Pharm. Sci..

[101] Wang D., Zhang S., Tang H., Jiang C., Wang B., Liu J. (2019). Development of sustained-release pellets to modulate the in vivo processes of the main active components of Danshen: A pharmacokinetic and pharmacodynamic evaluation. Phytomedicine.

[102] El-Moslemany R.M., El-Kamel A.H., Allam E.A., Khalifa H.M., Hussein A., Ashour A.A. (2022). Tanshinone IIA loaded bioactive nanoemulsion for alleviation of lipopolysaccharide induced acute lung injury via inhibition of endothelial glycocalyx shedding. Biomed. Pharmacother..

[103] Chen Y., Pan D., Zhu Q., Lu M., Zhang Y., Gao Z., Zhang L., Yi Y., Liu L., Liu Q. (2025). Biomimetic metal-phenolic nanocarrier for co-delivery of multiple phytomedical bioactive components for anti-atherosclerotic therapy. Int. J. Pharm..

[104] Chen X., Zhou Q., Wang B., Feng D., Jiang R., Wang X. (2024). Efficacy and safety of tanshinone IIA in combination with mesalazine in the treatment of ulcerative colitis: A Systematic review and meta-analysis. BMC Gastroenterol..

[105] Roth A., Zhao P., Soukup S.T., Guigas C., Stärke J., Kulling S.E., Diel P. (2023). Chemical Stability and Bioactivity of tanshinone I, tanshinone IIA, cryptotanshinone and dihydrotanshinone in in vitro test systems. Toxicol. Lett..

[106] Zhou H., Zhao Y., Peng W., Han W., Wang Z., Ren X., Wang D., Pan G., Lin Q., Wang X. (2021). Effect of Sodium Tanshinone IIA Sulfonate Injection on Blood Lipid in Patients With Coronary Heart Disease: A Systematic Review and Meta-Analysis of Randomized Clinical Trials. Front. Cardiovasc. Med..

[107] Morton J.S., Andersson I.J., Cheung P.-Y., Baker P., Davidge S.T., Torrens C. (2015). The vascular effects of sodium tanshinone IIA sulphonate in rodent and human pregnancy. PLoS ONE.

[108] Wu X., Fan M., Wei S., Guo D., Ong H.T. (2023). The efficacy and safety of sodium tanshinone ⅡA sulfonate injection in the treatment of unstable angina pectoris: A systematic review and meta-analysis. PLoS ONE.

[109] Tan D., Wu J.R., Zhang X.M., Liu S., Zhang B. (2018). Sodium Tanshinone II A Sulfonate Injection as Adjuvant Treatment for Unstable Angina Pectoris: A Meta-Analysis of 17 Randomized Controlled Trials. Chin. J. Integr. Med..

